# The Aryl hydrocarbon receptor mediates tobacco-induced PD-L1 expression and is associated with response to immunotherapy

**DOI:** 10.1038/s41467-019-08887-7

**Published:** 2019-03-08

**Authors:** Gui-Zhen Wang, Li Zhang, Xin-Chun Zhao, San-Hui Gao, Li-Wei Qu, Hong Yu, Wen-Feng Fang, Yong-Chun Zhou, Fan Liang, Chen Zhang, Yun-Chao Huang, Zhihua Liu, Yang-Xin Fu, Guang-Biao Zhou

**Affiliations:** 1 State Key Laboratory of Molecular Oncology, National Cancer Center/National Clinical Research Center for Cancer/Cancer Hospital, Chinese Academy of Medical Sciences and Peking Union Medical College, Beijing, 100021 China; 2 State Key Laboratory of Membrane Biology, Institute of Zoology, Chinese Academy of Sciences & University of Chinese Academy of Sciences, Beijing, 100101 China; 3 State Key Laboratory of Oncology in South China; Collaborative Innovation Center for Cancer Medicine; Medical Oncology Department, Sun Yat-Sen University Cancer Center, Guangzhou, 510060 China; 4 School of Chinese Materia Medica, Beijing University of Chinese Medicine, No. 11, Bei San Huan Dong Lu, Beijing, 100029 China; 5 Department of Thoracic Surgery, the Third Affiliated Hospital of Kunming Medical University (Yunnan Tumor Hospital), Kunming, 650106 China; 6 Department of Pathology, University of Texas Southwestern Medical Center, Dallas, TX 75390 USA

**Keywords:** Non-small-cell lung cancer, Non-small-cell lung cancer

## Abstract

Whether tobacco carcinogens enable exposed cells immune escape resulting in carcinogenesis, and why patients who smoke respond better to immunotherapies than non-smokers, remains poorly understood. Here we report that cigarette smoke and the carcinogen benzo(a)pyrene (BaP) induce PD-L1 expression on lung epithelial cells in vitro and in vivo, which is mediated by aryl hydrocarbon receptor (AhR). Anti-PD-L1 antibody or deficiency in *AhR* significantly suppresses BaP-induced lung cancer. In 37 patients treated with anti-PD-1 antibody pembrolizumab, 13/16 (81.3%) patients who achieve partial response or stable disease express high levels of AhR, whereas 12/16 (75%) patients with progression disease exhibit low levels of AhR in tumor tissues. AhR inhibitors exert significant antitumor activity and synergize with anti-PD-L1 antibody in lung cancer mouse models. These results demonstrate that tobacco smoke enables lung epithelial cells to escape from adaptive immunity to promote tumorigenesis, and AhR predicts the response to immunotherapy and represents an attractive therapeutic target.

## Abstract (web summary)

Lung cancer patients who smoke show a better response to immunotherapy than non-smokers. Here, the authors show that tobacco smoke induces PD-L1 expression on lung epithelial cells via AhR that is associated with benefits of PD-1 inhibitor in patients, shedding new lights on lung carcinogenesis and immunotherapy.

## Introduction

Tobacco smoke represents the single biggest public health threat the world is currently facing, killing around 7 million people a year^1^. More than 8000 compounds have been identified in tobacco and tobacco smoke, among which >70 ones are carcinogens. These include polycyclic aromatic hydrocarbons (PAHs), tobacco-specific nitrosamines, volatile nitrosamines, and many others^2^. Tobacco smoke induces a large amount of somatic genomic mutations in cancer tissues^3^ and counterpart normal controls^4,5^, and confers the exposed cells with the hallmarks of cancer^6–10^. However, whether and how the carcinogens render the exposed cells to escape the immune system to promote lung carcinogenesis, remains unclear.

Programmed cell death 1 ligand (PD-L1; also known as B7-H1, CD274) is an immune inhibitory receptor ligand that is expressed by cancer cells and cells in the tumor microenvironment^11,12^. Interaction of this ligand with its receptor programmed cell death receptor 1 (PD-1; or CD279) inhibits T-cell activation and cytokine production. PD-L1 is induced by cytokines such as interferon-γ (IFNγ)^13^ and oncogenes including epidermal growth factor receptor (EGFR)^14^, chimeric nucleophosmin (NPM)/anaplastic lymphoma kinase (ALK)^15^, transforming growth factor β (TGFβ)^16^, signal transducer and activator of transcription 3 (STAT3)^17^, and hypoxia inducible-factor-1α (HIF-1α)^18^. Amplification of 9p24.1^19^ and deficiency in phosphatase and tensin homolog (PTEN)^20^ or p53^21^ result in PD-L1 overexpression. Epigenetic modifiers and microRNAs also modulate PD-L1 expression^22,23^. However, the effect of environmental carcinogens on immune checkpoints needs to be elucidated.

PD-L1/PD-1 blockade therapy has yielded promising clinical responses in lung cancer patients^24–28^. As compared with nonsmoker patients, smoker patients receiving anti-PD-L1/PD-1 therapy exhibited improved objective response, durable clinical benefits, and progression-free survival^26,27^. By whole-exome sequencing of non–small cell lung cancers (NSCLCs) treated with a PD-1 antibody, Rizvi et al^29^ showed that the higher nonsynonymous mutation and higher neoantigen burden in tumors of smokers might contribute to improved response. The above results also suggest a possibility that smoking might induce a mechanism to suppress tumor specific T cell responses at early stage. We hypothesized that the carcinogens of tobacco smoke may modulate immune checkpoints and confer cancer cells immune escape. We tested this hypothesis in this study.

## Results

### Tobacco smoke induces PD-L1 expression on lung epithelial cells

**Fig. 1:**
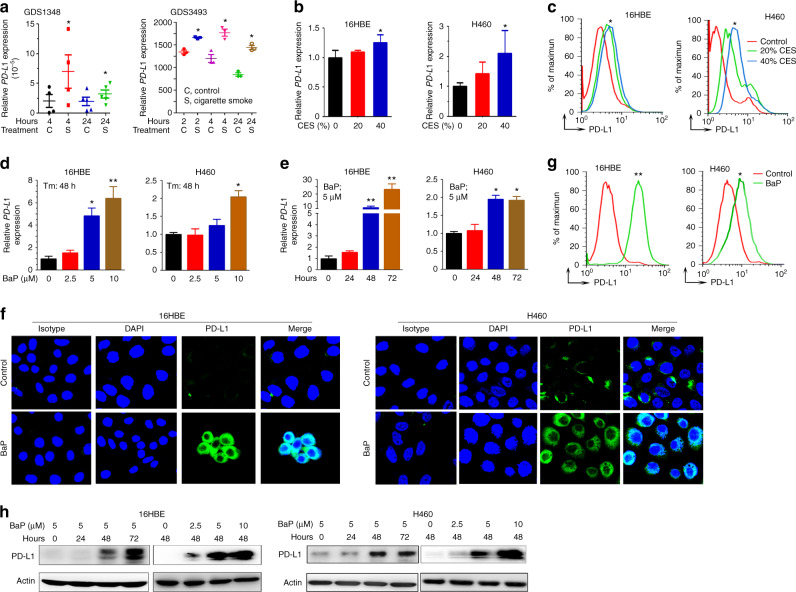
Tobacco smoke and carcinogen BaP induces PD-L1 expression on lung epithelial cells. **a** In microarray datasets of gene expression profiles of normal bronchial epithelial cells exposed to cigarette smoke, the expression of *PD-L1* was analyzed. C, control; S, cigarette smoke. Error bars, sd. **b**, **c** The cells were treated with cigarette smoke extract (CES) at indicated concentrations for 48 h, and the expression of PD-L1 was assessed by real-time RT-PCR (**b**) and flow cytometry (**c**). The experiments were conducted in triplicate and repeated for three times. Error bars, sd. **d**–**h** The cells were treated with BaP at indicated concentrations for indicated time points, and the expression of PD-L1 was assessed by real-time RT-PCR (**d**, **e**), immunofluorescence assays (**f**), flow cytometry (**g**), and western blot (**h**) assays. Student’s *t* test, *P < 0.05; **P < 0.01. Error bars, sd

We analyzed the immune checkpoint molecules in GDS1348 and GDS3493 microarray datasets of gene expression profiles of normal bronchial epithelial cells (http://www.ncbi.nlm.nih.gov/geo/), and reported that cigarette smoke significantly upregulated *PD-L1* in 2 to 24 h (Fig. 1a). Cigarette smoke extract (CES) was prepared^30^ and used to treat 16HBE (normal lung epithelial cells) and H460 (NSCLC) cells, and the results showed that treatment of the cells with 20 – 40% of CES significantly upregulated PD-L1 at both mRNA (Fig. 1b) and protein (Fig. 1c) levels.

We used the main tobacco carcinogens benzo(a)pyrene (BaP), nicotine-derived nitrosaminoketone (NNK), dibenz[a,h]anthracene (DbA) and benzo[g,h,i]perylene (BzP) to treat the cells, and showed that BaP upregulated *PD-L1* in a dose- and time-dependent manner (Fig. 1d, e). BaP increased PD-L1 at protein level, revealed by immunofluorescent (Fig. 1f), flow cytometry (Fig. 1g), and western blot (Fig. 1h) assays using an anti-PD-L1 antibody. DbA and BzP also upregulated *PD-L1* in 16HBE cells (supplementary Fig. 1), but the effects were much weaker than that induced by BaP. We therefore investigated the mechanisms of action of BaP-induced PD-L1 expression in this study.

### Tobacco smoke induces PD-L1 expression in vivo

**Fig. 2:**
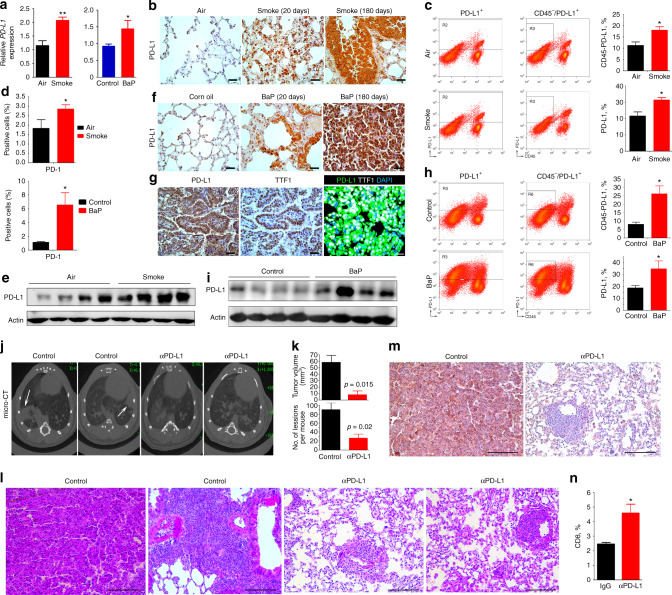
Tobacco smoke and BaP induce PD-L1 in mice, whereas inhibition of PD-L1 suppresses BaP-induced lung cancer. **a** The A/J mice were exposed to tobacco smoke or treated with BaP for 20 days and sacrificed, the expression *PD-L1* in lung tissues was detected by real-time PCR. **b** The lung tissues were isolated and analyzed by immunohistochemistry using an anti-PD-L1 antibody. Scale bar = 2000 μm. **c, d** Flow cytometry analysis of PD-L1^+^, CD45^-^PD-L1^+^ cells (**c**) and PD-1^+^ (**d**) cells in lung tissues of mice exposed to tobacco smoke. PD-1 + cells in lung tissues of mice treated with BaP were also shown (**d**). PD-1^+^ cells in this experiment were analyzed from the CD45^+^CD3^+^ leukocytes isolated from the same tumor. **e** The expression of PD-L1 in lung tissues of mice exposed to tobacco smoke was assayed by Western blot. **f** Lung tissues of BaP-treated mice were isolated and analyzed by IHC assays using an anti-PD-L1 antibody. Scale bar = 2000 μm. **g** IHC (left and middle panels) and immunofluorescence (right) assays of lung tissues of BaP-treated mice using continuous sections of 5 μm. IHC assay was conducted using anti-TTF1 and anti-PD-L1 antibodies, and immunofluorescence assay was performed using anti-PD-L1 (green) and anti-TTF1 (white) antibodies, and 4,6-diamidino-2-phenylindole (DAPI) to stain the nucleus (blue). IHC assays, scale bar = 2000 μm; immunofluorescence assays, scale bar = 20 μm. **h** Flow cytometry analysis of PD-L1^+^ and CD45^-^PD-L1^+^ cells in lung tissues of mice treated with BaP. **i** The expression of PD-L1 in lung tissues of BaP-treated mice was assayed by Western blot. **j** Micro-CT scanning of lungs of BaP-treated mice with or without anti-PD-L1 antibody. **k** Comparison of tumors in mice of the two groups. **l** Hematoxylin-eosin (HE) staining of lung sections from BaP-treated mice with or without PD-L1 blockade. Lesions/mouse are shown in **(k)**. Scale bar = 500 μm. **m** IHC assays of lung tumor tissues of BaP-treated mice using an anti-PD-L1 antibody. Scale bar = 500 μm. **n** Flow cytometry analysis of CD45^+^CD3^+^CD8^+^ (CD8^+^) cells in tumor tissues of mice treated with BaP and anti-PD-L1 antibody. Student’s *t* test, *P < 0.05; **P < 0.01. Error bars, sd

The A/J mice (n = 10 for each group) were exposed to cigarette smoke with filtered conditioned air of 750 µg total particulate matter (TPM) per liter (TPM/l) for up to 12 months^31^ and PD-L1 in lung tissues was measured. We found that as compared with mice exposed to clean air, mice exposed to cigarette smoke had higher *PD-L1* in their lung tissues (Fig. 2a, left panel). The membranous PD-L1 on lung epithelial cells was upregulated, revealed by immunohistochemistry (IHC) staining (Fig. 2b). Flow cytometry analysis showed that the total PD-L1^+^ and CD45^-^/PD-L1^+^ cells were increased by tobacco smoke in cell populations prepared from lung tissues (Fig. 2c). PD-1^+^ T lymphocytes were also increased in lung tissues of mice exposed to tobacco smoke (Fig. 2d, upper panel). The lysates of lung tissues were subjected to Western blot and the results showed that tobacco induced upregulation of PD-L1 in mice (Fig. 2e).

In mice (n = 10 for each group) treated with BaP at 100 mg/kg twice a week for 5 weeks^10^, the expression of *PD-L1* at mRNA level was upregulated (Fig. 2a, right panel). BaP induced upregulation of PD-L1 on cell membrane of lung tissues (Fig. 2f). IHC staining showed that PD-L1 positive cells were mainly TTF1-positive cancer cells (Fig. 2g). Total PD-L1^+^ and CD45^-^/PD-L1^+^ cells (Fig. 2h) as well as PD-1^+^ cells (Fig. 2d, lower panel) were increased in cell populations prepared from lung tissues of the mice. Western blot analysis of lung tissue lysates confirmed the upregulation of PD-L1 by BaP (Fig. 2i). These results indicate that tobacco smoke and BaP induce PD-L1 on lung epithelial cells in vivo.

### Inhibition of PD-L1 suppresses BaP-induced lung cancer

We tested the effect of PD-L1 blockade in A/J mice (*n* = 10 for each group) treated with BaP^10^ in the presence or absence of anti-PD-L1 antibody (200 µg once a week for 5 weeks). Six months later, the mice were scanned by microscopic computed tomography (micro-CT) and the results showed that while BaP induced lung cancer, PD-L1 blockade effectively suppressed tumor formation (Fig. 2j, k). Hematoxylin-eosin (HE) staining of the tissues demonstrated that PD-L1 blockade-treated mice have much reduced area of lung adenocarcinoma (Fig. 2l) and less lesions (Fig. 2k) and decreased PD-L1^+^ lung cancer cells (Fig. 2m). Previous studies showed that sufficient T cell infiltration is essential for response to PD-L1 blockade^32^. By flow cytometry analysis, we showed that the CD45^+^CD3^+^CD8^+^ cells in mice treated with PD-L1 blockade were significantly increased (Fig. 2n).

### Aryl hydrocarbon receptor-mediates tobacco-induced PD-L1

**Fig. 3:**
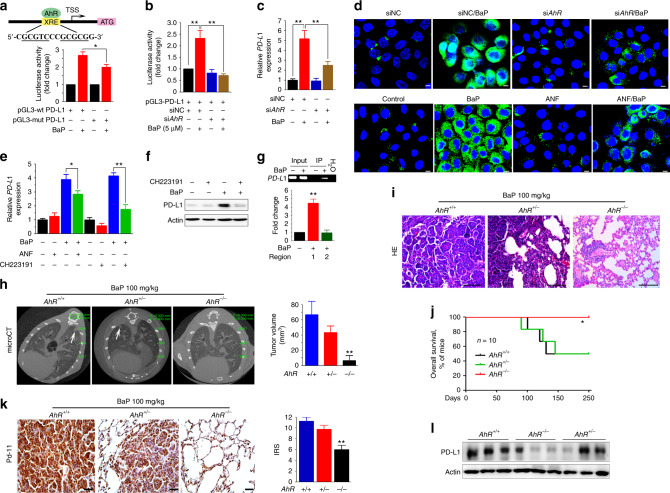
AhR mediates BaP-induced PD-L1 expression. **a** The AhR binding site of *PD-L1* promoter (upper panel). TSS, transcription start site. The H460 cells were transfected with the wild-type (wt) or mutant (mut; deletion of 5’-GCGTC-3’ fragment in the XRE-like sequence) *PD-L1* promoter-luciferase reporter construct, treated with BaP for 48 h, and assessed by the luciferase assays (lower panel). Student’s *t* test, *, *P* < 0.05; **, *P* < 0.01. Error bars, sd. **b** Luciferase assays were performed in H460 cells transfected with wt *PD-L1* promoter-luciferase reporter construct and siRNAs in the absence or presence of BaP. **c**, **d** The expression of PD-L1 at mRNA (detected by real-time PCR, **c)** and protein (detected by immunofluorescence assay, (**d**) levels in *AhR* silencing 16HBE cells upon BaP. Scale bar = 20 μm. **e** The expression of *PD-L1* at mRNA (detected by real-time PCR) level in 16HBE cells co-incubated with BaP, with or without ANF/CH223191 treatment. **f** Western blot analysis of PD-L1 in 16HBE cells treated with CH223191 (10 μM) and/or BaP (5 μM) for 48 h. **g** Chromatin immunoprecipitation (ChIP) assay was performed using AhR-precipitated DNA samples of 16HBE cells (treated with or without BaP) and primers for *PD-L1*. The expression of PD-L1 was evaluated by RT-PCR (upper panel) and real-time RT-PCR (lower panel). **h** AhR deficient mice were treated with BaP, scanned by Micro-CT, and the tumor volume was calculated. **i** HE staining of lung tissue sections from *AhR* wild-type (wt) or knockout mice. Scale bar = 500 μm. **j** Lifespan of the mice. Log-rank test, *P < 0.05; **P < 0.01. **k** Immunohistochemistry assays and the corresponding immunoreactivity score (IRS) of lung tissue sections using an anti-PD-L1 antibody. Scale bar = 2000 μm. **l** Western blot analysis of lysates of lung tissues from AhR deficient mice treated with BaP

Aryl hydrocarbon receptor (AhR) mediates BaP-induced production of chemokine CXCL13 by lung epithelial cells^10^, and is critical to BaP-induced skin cancer^33^. By analyzing the sequence of *PD-L1* gene, we found two XRE-like elements, 5’-GCGTC-3’ and 5’-GCGCG-3’, in its promoter region (Fig. 3a). While BaP increased wild-type (wt) *PD-L1* promoter-driven luciferase activity, deletion of one XRE-like element (5’-GCGTC-3’) significantly attenuated this activity (Fig. 3a). In in vitro reporter assays, silencing of *AhR* significantly suppressed BaP-induced *PD-L1* in the cells (Fig. 3b). In 16HBE cells upon BaP, PD-L1 was inhibited by si*AhR* at both mRNA and protein levels detected by quantitative RT-PCR (Fig. 3c) and immunofluorescence analysis (Fig. 3d, upper panel). A synthetic flavone derivative AhR antagonist, alpha-naphthoflavone (ANF)^34^, inhibited BaP-induced PD-L1 at both mRNA (Fig. 3e) and protein (Fig. 3d, lower panel) levels. Since ANF also bears agonistic effect on AhR in that it can activate AhR target *CYP1A1* promoter^35^, a more specific AhR antagonist CH223191^36^ was further tested. We showed that CH223191 suppressed BaP-induced PD-L1 at both mRNA (Fig. 3e) and protein (Fig. 3f) levels at a stronger extent than ANF. Chromatin immunoprecipitation (ChIP) and RT-PCR/qRT-PCR assays were conducted to test AhR-*PD-L1* interaction, and the results showed that AhR directly bound the promoter of *PD-L1* at -700 to -100 (region 1) but not other region (Fig. 3g).

To further test the role AhR plays in BaP-induced PD-L1 expression, the *AhR*-deficient mice^37^ were treated with BaP and tested by micro-CT. We found that in *AhR*^+/+^ mice, BaP induced lung cancer (Fig. 3h); but in *AhR*^-/-^ mice, BaP-induced lung cancer was inhibited (Fig. 3h, i), life span was prolonged (Fig. 3j), and PD-L1 upregulation was markedly attenuated (Fig. 3k, l). These data indicate that AhR is critical to PD-L1 expression.

### Smokers have higher PD-L1 expression than nonsmokers

**Fig. 4:**
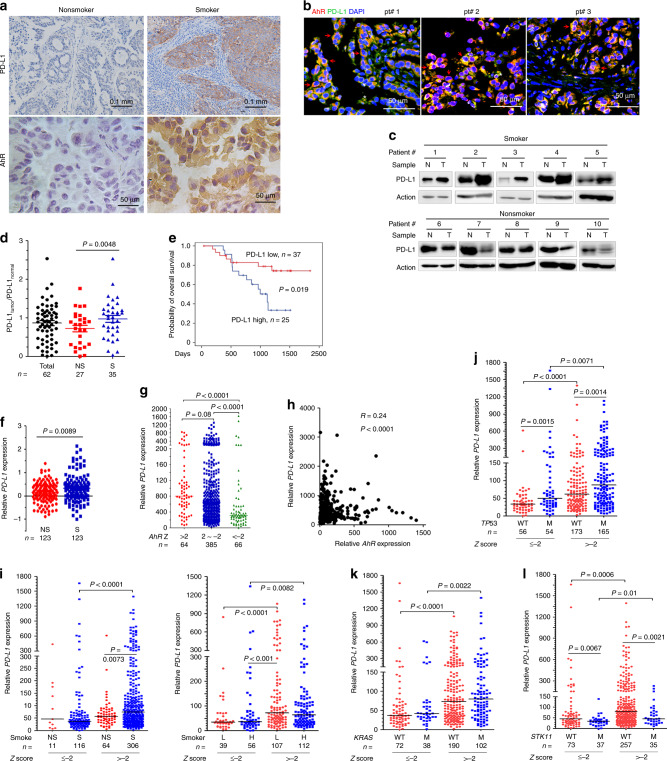
A threshold level of *AhR* is critical to increased *PD-L1* expression. **a** The expression of PD-L1 and AhR was detected by IHC assay in NSCLCs. **b** Immunofluorescence assays of smoker patients’ formalin-fixed paraffin embedded (FFPE) 5-μm sections using antibodies against PD-L1 and AhR, and DAPI. Arrow, cancer cells; *, non-cancerous cells. **c** Western blot analyses of lysates of tumor (T) and adjacent normal (N) lung tissues harvested from NSCLCs (n = 62). **d** Quantification of the ratios of PD-L1 in tumors to PD-L1 in counterpart normal lung tissues in smoker and nonsmoker patients. Determined by densitometry analyses of immunoblot bands in (**c**). NS, nonsmoker; S, smoker. *P* value, Student’s *t* test. **e** Overall survival of the 62 patients. *P* value, log-rank test. **f** PD-L1 expression in an Oncomine report. **g** The expression of *PD-L1* and *AhR* in DNA microarray data of TCGA datasets. *AhR* z score was calculated as: (RNA-Seq by Expectation Maximization (RESM) in tumor - mean of RESM values in normal)/Standard deviation of RESM values in normal). *P* value, Mann-Whitney test. **h** The association between *PD-L1* and *AhR* in patients. Data are from TCGA datasets. **i** The smoking status, *PD-L1* expression, and *AhR* Z score in NSCLCs. **j**–**l** The expression of *PD-L1*, *AhR* Z score, and *TP53* (**j**), *KRAS* (**k**), or *STK11* (**l**). *P* values in i-l, Mann-Whitney test. WT wild type, M mutant, H heavy smoker, L light smoker

We tested the expression of PD-L1 in tumor tissues of 62 patients by IHC staining, and found that smokers had higher membranous PD-L1 on tumor cells than nonsmokers (Fig. 4a). AhR expression in smoker patients was also higher than in nonsmoker patients (Fig. 4a). To investigate whether AhR and PD-L1 co-localize in specific cells in the tumor microenvironment, immunofluorescent assays were performed using antibodies against AhR and PD-L1, and DAPI. The staining pattern was analyzed in combination with the morphology of the cells. We reported that cancer cells expressed both AhR and PD-L1 at a relatively high level, which represent the main cells on which AhR and PD-L1 were co-localized (arrow, Fig. 4b). There are some non-cancerous cells that also co-expressed AhR and PD-L1 (Fig. 4b), which morphologically exhibited lymphocyte characteristics. Western blot analyses (Fig. 4c) and the ratios of PD-L1 in tumors to PD-L1 in counterpart normal controls (determined by densitometry analyses of immunoblot bands) (Fig. 4d) showed that smokers had higher PD-L1 than nonsmokers. Among the 35 smokers, 18 (51.4%) patients had higher PD-L1 in tumor tissues than in normal tissues, whereas 7/27 (25.9%) nonsmokers had higher expression of PD-L1 in tumor tissues (Table 1). Without anti-PD-L1/anti-PD-1 treatment, patients with higher PD-L1 had shorter overall survival than patients with lower PD-L1 (Fig. 4e).

**Table 1:** Baseline demographic characteristics of the patients

Characteristics	Case, n	PD-L1-high, n (%)	*P* value*
Total	62	25 (40.3)	
Gender			
Male	41	20 (48.8)	0.06
Female	21	5 (23.8)	
Smoking			
Smoker	35	18 (51.4)	0.04
Non-smoker	27	7 (25.9)	
Age			
<65	46	20 (43.5)	0.39
≥65	16	5 (31.2)	
Histology			
Adenocarcinoma	38	14 (36.8)	0.54
Squamous-cell carcinoma	20	9 (45)	
Others	4	2 (50)	
TNM stage			
I	28	10 (35.6)	0.3
II	7	2 (28.6)	
III	22	10 (45.5)	
IV	5	3 (60)	

* tested by the Fisher exact test.

### A threshold level of *AhR* is critical to *PD-L1* overexpression

To investigate *PD-L1* expression in NSCLCs of other cohorts, a cancer microarray database Oncomine (www.oncomine.org) was applied. In a cohort of 123 smokers and 123 nonsmokers^38^, the smoker patients had higher *PD-L1* than nonsmokers (Student’s *t* test, *P* = 0.0089; Fig. 4f). The DNA microarray data of 515 patients were downloaded from the Cancer Genomics Hub (CGHub) (https://cghub.ucsc.edu/) of The Cancer Genome Atlas (TCGA) datasets, and the association of *PD-L1* expression and *AhR* Z score was tested. We found that the relative *PD-L1* expression in patients with *AhR* Z score of ≥ −2 was much higher than patients with *AhR* Z score < −2 (Fig. 4g), and *PD-L1* expression was associated with *AhR* expression (Fig. 4h). In patients with *AhR* Z score ≤ −2, the difference in *PD-L1* between smokers and nonsmokers was not statistically significant, but in patients with *AhR* Z score > −2, the difference was statistically significant (Mann-Whitney test, *P* = 0.0073; Fig. 4i). Smokers with *AhR* Z score > −2 had much higher *PD-L1* than smokers with *AhR* Z score ≤ −2 (Fig. 4i, left panel). Heavy ( ≥ 30 pack years) and light ( < 30 pack years) smokers with *AhR* Z score > −2 had much higher *PD-L1* than patients with *AhR* Z score ≤ −2, and light smokers with *AhR* Z score > −2 had higher *PD-L1* than heavy smokers with *AhR* Z score ≤ −2 (Fig. 4i, light panel).

We analyzed the association of *PD-L1* expression and some driver mutations, and found that *PD-L1* in patients with mutant *TP53* was higher than patients with wild type (WT) *TP53*, but *PD-L1* in cases with *AhR* Z score > −2 and WT *TP53* was higher than in patients with *AhR* Z score ≤ −2 and mutant *TP53* (Mann-Whitney test, *P* = 0.02; Fig. 4j). In patients with WT or mutant *KRAS*, those with *AhR* Z score of > −2 had higher *PD-L1* than patients with *AhR* Z score of ≤ −2 (Fig. 4k). Patients with WT *STK11* had higher *PD-L1* than patients with mutant *STK11*, and patients with higher *AhR* Z score had higher *PD-L1*; but in patients with WT *STK11* and *AhR* Z score of ≤ −2, *PD-L1* was not higher than in patients with mutant *STK11* and *AhR* Z score > −2 (Fig. 4l). These results suggested an important role of *AhR* in determining *PD-L1* expression level.

### AhR is associated with clinical benefit of anti–PD-1 therapy

**Fig. 5:**
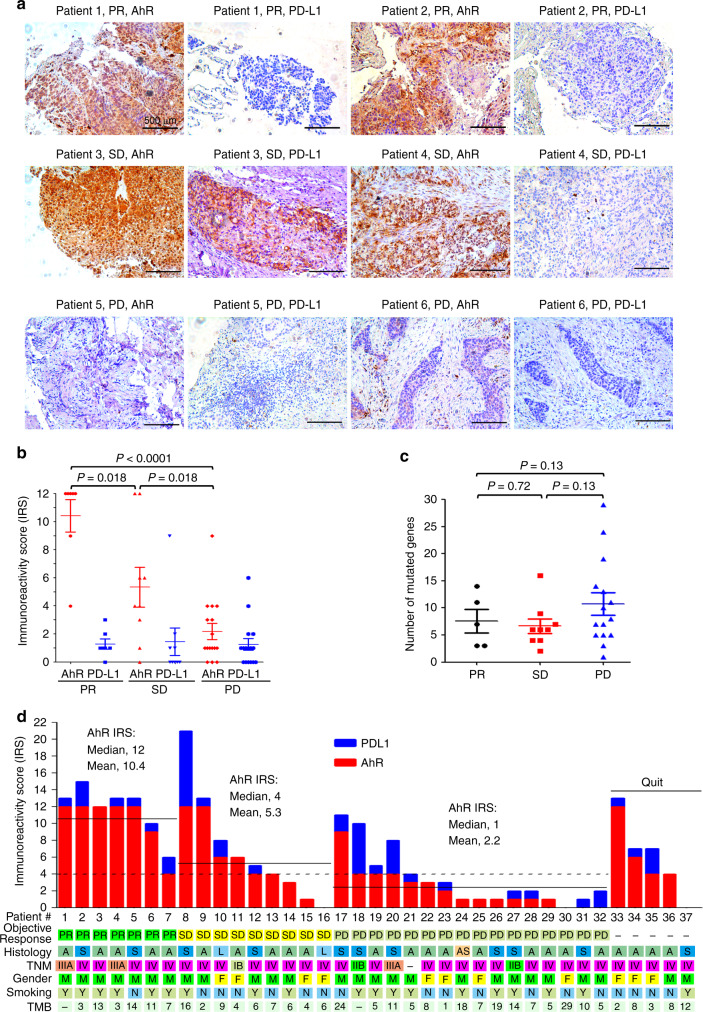
AhR expression is associated with clinical benefits of PD-1 blockade. **a** The expression of AhR and PD-L1 (detected by IHC) and response of the patients to pembrolizumab. **b** IRS of AhR and PD-L1 in patients achieved PR, SD, or PD. **c** A total of 422 genes (supplementary Table 1) were sequenced in 35 out of the 37 patients, and the number of mutated genes per sample are shown. Error bars, sd. *P* values in b, c, Student’s *t* test. **d** Clinical characteristics, AhR/PD-L1 IRS, TMB, and response of the patients to pembrolizumab. Objective response: PR partial response, SD stable disease, PD progression of disease. Histology: A adenocarcinoma, AS adenosquamous carcinoma, L lymphoepithelioma-like carcinoma of the lung, S squamous cell carcinoma. Gender: F female, M male. Smoking: Y yes, N no

We tested the potential association between the AhR expression and clinical outcome of patients treated with anti-PD-1 antibody pembrolizumab. To do this, 37 NSCLCs previously treated with cisplatin-based chemotherapies (Table 2) were enrolled, and IHC was conducted to detect the expression of AhR and PD-L1 on tumors and counterpart normal lung tissues. The patients were then treated with pembrolizumab as described^27^. We found that AhR staining on tumors of patients achieved partial response (PR) and stable disease (SD) was much stronger than patients with progression of disease (PD) (Fig. 5a), and IRS of patients achieved PR and SD was significantly higher than cases with PD (Fig. 5b, Table 2). The multivariate logistic analyses showed that AhR-high was associated with beneficial effect of pembrolizumab (Table 3). In this setting, the expression of PD-L1 on cancer cells could not predict responses to pembrolizumab, in that some tumors expressed PD-L1 did not respond, and some responses occurred in PD-L1–negative tumors (Fig. 5a, b), in consistence with a previous report^12^.

**Table 2:** Clinical Characteristics of the patients administrated with pembrolizumab

Characteristics	Case, n	PR, n	SD, n	PD, n	*P*-value
Gender					
Male	26	7	5	12	1
Female	11		4	4	
Smoking					
Smoker	19	6	3	9	1
Non-smoker	18	1	6	7	
Age					
<65	31	5	7	14	0.36
≥65	6	2	2	2	
Histology					
Adenocarcinoma	23	5	5	9	0.52
Squamous-cell carcinoma	11	2	2	6	
Others	3		2	1	
TNM stage					
I	1		1		0.93
II	2			2	
III	3	2		1	
IV	30	5	8	12	
Unknown	1			1	
AhR IRS					
≥4	21	7	6	4	0.001
<4	16	0	3	12	
No. of TMB					
≥7	19	3	3	10	0.20
<7	15	2	6	5	
Unknown	3	2		1	

*PR* partial response, *SD* stable disease, *PD* progression of disease, *P* value patients achieved PR and SD versus cases with PD

**Table 3:** AhR-high and patients’ clinical characteristics

Variable	Odds ratio	95% confidence interval	*P* values
Gender	5.422	0.412–71.339	0.199
Age	1.094	0.011–112.872	0.970
Smoking	5.889	0.288–120.5	0.250
Histology	0.957	0.322–2.840	0.937
TNM stage	1.356	0.495–3.714	0.553
Beneficial effect	14.568	2.530–83.896	0.003
PD-L1	6.520E9	0	0.999
TMB	2.646	0.160-43.869	0.497

The association between AhR-high and clinical characteristics of the 37 patients treated with pembrolizumab was analyzed by multivariate logistic analyses

Smokers have more somatic mutations than non-smoker patients^39^, raising the possibility that mutation burdens may link to AhR levels. We tested this possibility by analyzing the mutation loads of LUADs, LUSCs, and other cancers such as esophageal carcinomas (ESCAs) and stomach adenocarcinomas (STADs) in TCGA datasets, and reported that while LUADs, LUSCs, and STADs had more mutations/tumor than ESCAs (supplementary Fig. 2a), AhR expression levels in ESCAs were higher than LUADs, LUSCs, and STADs (supplementary Fig. 2b). These results indicate that AhR levels are not associated with mutation burdens of the patients. To further investigate the tumor mutation burden (TMB) of the patients treated with pembrolizumab, a total of 422 genes (Supplementary Table 1) were sequenced in tumor samples of 35 of the 37 patient, and our results showed that the patients harbored 1 – 29 (median, 7) mutated genes (Fig. 5c, d and Table 2). Multivariate logistic analyses showed that in this setting, TMB was not associated with clinical outcome of the patients treated with pembrolizumab (Fig. 5c, d and Tables 2, 3). Hence, AhR may be an independent factor for clinical outcome of patients upon pembrolizumab treatment.

### AhR modulator exhibits anti-lung cancer activity in vivo

**Fig. 6:**
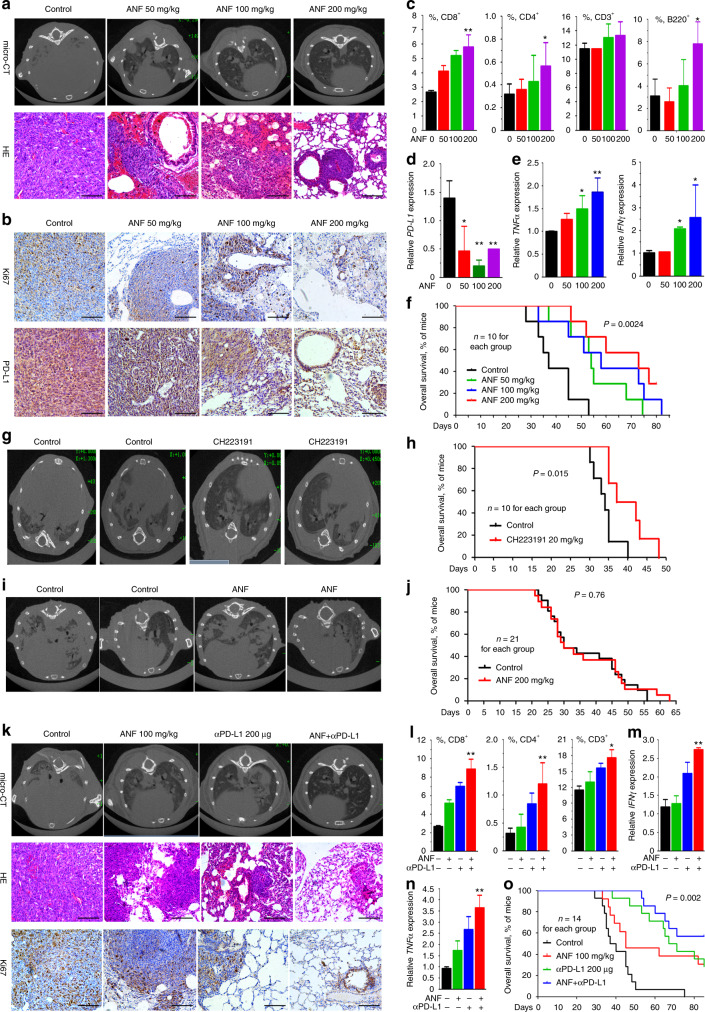
AhR inhibitor exhibits anti-lung cancer activity in vivo. **a** C57BL/6 mice were intravenously injected with LLC (5 × 10^5^) cells, and 3 days later the mice were randomized to receive vehicle or ANF treatment. Micro-CT scanning images and HE staining of lung sections are shown. Scale bar = 500 μm. **b** IHC assays of ANF-treated mice’ lung tumor tissues using indicated antibodies. Scale bar = 500 μm. **c** Flow cytometry analysis of CD8^+^, CD4^+^, CD45^+^CD3^+^, and CD45^+^B220^+^ cells in the lung tissues. **d** The expression of *PD-L1* in lung tissues was detected by real-time PCR. **e** The expression of *TNFα* and *IFNγ* in the lung tissues was detected by real-time PCR. **f** Life span of the mice. **g**, **h** The C57BL/6 mice were intravenously injected with LLC (5 × 10^5^) cells, and 3 days later randomized to receive vehicle or CH223191 treatment. Micro-CT scanning images (**g**) and life span of the mice (**h**) are shown. **i**, **j** The NOD/SCID mice were intravenously injected with LLC (5 × 10^5^) cells, and 3 days later randomized to receive vehicle or ANF treatment. Micro-CT scanning images (**i**) and life span of the mice (**j**) are shown. **k** C57BL/6 mice were injected intravenously with LLC (5 × 10^5^) cells and treated with ANF and/or anti-PD-L1 antibody. Micro-CT scanning images, HE and IHC staining of lung sections of the mice are shown. Scale bar = 500 μm. **l** Flow cytometry analysis of CD8^+^, CD4^+^, and CD3^+^ cells in the lung tissues. **m**, **n** The expression of *IFNγ* (**m**) and *TNFα* (**n**) in the lung tissues was detected by real-time PCR. **o** Life span of the mice. *P* values in c-e, l-n, Student’s t test; *P* values in f, h, j, o, Log-rank test. Error bars, sd

We tested the efficacy of ANF (50 to 200 mg/(kg·day) for 22 days) in C57BL mice harboring 5 × 10^5^ Lewis lung carcinoma (LLC) cells that express high level of PD-L1^18^. Micro-CT results showed disseminated disease in both lungs of the mice treated with vehicle control, whereas ANF markedly suppressed tumor growth (Fig. 6a and supplementary Fig. 3a). Histologic examination showed a clearly decreased cellularity and reduced tumor load in lungs of ANF-treated mice compared with that of control mice (Fig. 6a). ANF downregulated the expression of proliferation index Ki67 (Fig. 6b), reduced PD-L1^+^ cells (Fig. 6b), and increased CD8^+^, CD4^+^, CD3^+^, and B220^+^ cells (Fig. 6c) in the lungs. ANF downregulated PD-L1 (Fig. 6b, d) and upregulated tumor necrosis factor α (*TNFα*) and interferon γ (*IFNγ*) in lungs of mice injected with LLC cells (Fig. 6e). ANF significantly prolonged life span (*P* = 0.0024; Fig. 6f) but did not perturb the body weight (supplementary Fig. 3b) of mice bearing LLC cells. CH223191 also markedly suppressed disseminated disease (Fig. 6g) and significantly prolonged life span (Fig. 6h) of the LLC cells-harboring C57BL mice at a relatively low dosage (20 mg/kg). However, ANF did not significantly inhibited tumor growth (Fig. 6i) or prolong overall survival (Fig. 6j) of immune compromised NOD/SCID mice inoculated with LLC cells, suggesting that the immune modulation activity is important to ANF’s anti-lung cancer activity.

### ANF enhances PD-L1 antibody in treating murine lung cancer

We further showed that while anti-PD-L1 antibody exhibited anti-lung cancer activity, ANF significantly enhanced its efficacy in LLC cells-harboring mice, reflected by micro-CT, histologic examination, and Ki67 staining (Fig. 6k). Combined use of ANF and anti-PD-L1 antibody significantly increased infiltration of CD8^+^, CD4^+^, and CD3^+^ T lymphocytes into tumor tissues (Fig. 6l), and upregulated the expression of *IFNγ* (Fig. 6m) and *TNFα* (Fig. 6n). Kaplan-Meier analysis showed that combination of ANF and anti-PD-L1 antibody significantly prolonged the lifespan of the mice (Fig. 6o). Treatment with ANF/anti-PD-L1 antibody did not reduce the body weight of the mice (supplementary Fig. 3c).

### ANF in treating colon cancer and fibrosarcoma murine models

We showed that in C57BL/6 mice subcutaneously injected with PD-L1-expressing MC38^32^ murine colon cancer cells (5 × 10^5^), ANF significantly suppressed tumor growth and potentiated the efficacy of anti-PD-L1 antibody (Supplementary Fig. 4a-c). ANF inhibited Ki67 expression and induced apoptosis of tumor cells reflected by Cleaved Caspase-3 (Casp-3) upregulation (Supplementary Fig. 4d). ANF also enhanced the effects of anti-PD-L1 antibody on Ki67 and Cleaved Casp-3 expression (Supplementary Fig. 4d). ANF in combination with PD-L1 blockade exerted potentiated effects in induction of infiltration of CD3, CD8, CD4, and B220 positive cells into tumor tissues (Supplementary Fig. 4e), and upregulation of *IFNγ* and *TNFα* expression by the lungs (Supplementary Fig. 4f). ANF/anti-PD-L1 antibody did not reduce the body weight of the mice (Supplementary Fig. 4g). In mice harboring Ag104Ld fibrosarcoma cells that are resistant to immune checkpoint inhibitor^32^, treatment with ANF/anti-PD-L1 antibody significantly inhibited tumor growth (Supplementary Fig. 5a) and increased infiltration of CD8^+^ and CD3^+^ T lymphocytes into the tumors (Supplementary Fig. 5b).

## Discussion

More than 87% of the lung cancer deaths are caused by cigarette smoke^40^. However, the detailed tobacco-induced lung carcinogenesis remains to be elucidated. Here, we reported that tobacco smoke and related carcinogens induced PD-L1 expression in normal and cancerous lung epithelial cells in vitro (Fig. 1) and in TTF1 positive lung epithelial cells in mice (Fig. 2). The expression of PD-L1 and AhR in lung cancer tissues of smoker patients was higher than in nonsmoker patients, and these molecules co-localize on lung cancer cells (Fig. 4). Indeed, tobacco smoke and BaP induced enrichment of CD4^+^, CD8^+^, and PD-1^+^ lymphocytes in lungs of the mice (Fig. 2), probably suggesting the compensation of immune system to suppress malignant transformation. However, lung cancer cells expressing PD-L1 may evade immunosurveillance by engaging the PD-1 immune checkpoint^41^. Therefore, tobacco smoke confers lung cancer cells evasion of immune destruction via induction of PD-L1 onto lung epithelial cells.

Antibodies against PD-L1 significantly improve progression-free survival and overall survival of a proportion of patients with most subtypes of cancer^42,43^. However, whether checkpoint inhibitors have a role in prevention of cancer formation remains largely unknown. Since tobacco smoke induced the expression of PD-L1 in lungs at an early stage, i.e., 20 days after the first exposure (Fig. 2), we hypothesized that suppression of PD-L1 may prevent formation of lung cancer. To test this possibility, the mice were treated with anti-PD-L1 antibody when they were exposed to BaP. Interestingly, treatment with PD-L1 antibody at 200 µg once a week for 5 weeks significantly inhibited BaP-induced lung cancer detected by micro-CT 6 months later (Fig. 2). In histologic sections, large tumors and disseminated cancer cells were easily found in control mice, but only limited and small tumor nodules were seen in anti-PD-L1 antibody-treated mice (Fig. 2). Though further evidence, e.g., treatment with longer terms or higher doses of anti-PD-L1 antibody in mice exposed to tobacco smoke, is needed, our results demonstrate that antagonizing PD-L1 may be helpful for prevention of lung cancer in smokers.

AhR is a basic helix-loop-helix transcription factors which binds the xenobiotic-responsive element (XRE) or aryl hydrocarbon response element (AHRE) to regulate target genes in response to PAHs and dioxin^44^. AhR is expressed in all tissues, and plays an important role in modulation of immune response^45^. AhR is critical to BaP-induced skin cancer^33^. We recently showed that AhR mediates BaP-induced production of a chemokine CXCL13, knockout of which significantly inhibits BaP-initiated lung cancer^10^. Here, we reported that PD-L1 was also a target of AhR, and the expression level of AhR was associated with PD-L1 expression in human NSCLCs (Fig. 4). PD-L1 was crucial for BaP-induced lung cancer, since blockade of this immune checkpoint suppressed BaP-triggered lung carcinogenesis (Fig. 2). These results indicate the critical role of AhR in environmental lung tumorigenesis. Indeed, deficiency in *AhR* abrogated BaP-caused lung cancer (Fig. 3). AhR is also required for kynurenine-induced PD-L1 in T cells^46^. AhR may also act through PD-L1 independent mechanisms in lung cancer cells. Hence, AhR has a central role in lung carcinogenesis and may serve as a target for chemoprevention and treatment of lung cancer.

Identification of predictive companion biomarkers represents one of the biggest challenges for clinical application of immune checkpoint inhibitors. PD-L1 expression on cancer cells cannot precisely predict response^12^, because many tumors expressed PD-L1 do not respond, and some responses occur in PD-L1–negative tumors. Tumor mutational burden^29^ and mismatch repair deficiency^47^ predict a proportion of responders. Here, we showed that AhR expression might have a role in prediction of patients’ responses to pembrolizumab, because patients achieved PR had much higher AhR than those with SD, and SD cases exhibited significantly higher AhR than patients with PD (Fig. 5). Of the 16 patients achieve PR and SD, only 3 (18.75%) cases had AhR IRS less than 4; of the 16 NSCLCs with PD, only 1 (6.25%) patient expressed AhR with IRS greater than 4 (Fig. 5). In these patients, multivariate logistic analyses showed that TMB was not associated with clinical outcome (Fig. 5 and Tables 2, 3).Some nonsmokers expressed high level of AhR and response to pembrolizumab, possibly reflecting their exposure to PAHs in different environmental media (such as air, soil, and water) and foods^48,49^. Future works are warranted to expand these observations and determine the significance of AhR in prediction of patients’ response to immune checkpoint inhibitors, alone or in combination with other biomarkers.

Limitations of immune-checkpoint inhibitors include the fact that only a proportion (20-30%) of patients benefited from these expensive therapeutics and the development of drug resistance mainly mediated by *JAK1*/*JAK2* mutations^50^ and type I and II interferons^51^. We found that in murine lung cancer model, ANF induced infiltration of CD8^+^ T lymphocytes into tumor tissues and increased *IFNγ* and *TNFα* expression, suppressed lung cancer cell proliferation, prolonged life-span of the mice, and significantly enhanced the therapeutic efficacy of anti-PD-L1 antibody (Fig. 6). In mouse models for PD-L1 blockade-sensitive colon cancer and PD-L1 blockade-resistant fibrosarcoma^32^, combined use of ANF and anti-PD-L1 antibody resulted in potentiated T cell infiltration and increased *IFNγ* and *TNFα* expression, as well as significant tumor growth inhibition (supplementary Fig. 4, 5). A more specific AhR antagonist CH223191 also showed therapeutic efficacy on C57 mice bearing LLC cells (Fig. 6). These results indicate that inhibition of AhR-PD-L1 axis and inhibition of AhR in immune cells pave the way to anti-lung cancer efficacy of AhR inhibitor, and the ANF/anti-PD-L1 antibody combination represents a novel strategy of cancer immunotherapy.

## Methods

### Patients

The study was approved by the research ethics committees of the Sun Yat-Sen University Cancer Center, and the Third Affiliated Hospital of Kunming Medical University (Yunnan Tumor Hospital). All lung cancer samples were collected with informed consent. Fresh tumor samples and counterpart normal lung tissues of 62 previously untreated NSCLCs (Table 1) were collected and tested by IHC and Western blot for the expression of interest targets. Moreover, 37 NSCLCs (Table 2) previously treated with cisplatin-based chemotherapies were enrolled, tumor biopsy specimens were collected and analyzed by IHC using an anti-AhR antibody and mouse monoclonal anti-human PD-L1 antibody PD-L1 IHC 28-8 pharmDx (Dako, Carpinteria, CA, USA), and received pembrolizumab (Keytruda, Merck, Kenilworth, NJ, USA) at a dose of 2 mg per kilogram of body weight every 3 weeks (ClinicalTrials.gov number, NCT02835690). Five patients quit pembrolizumab due to grade 4 rash, sudden death, or stroke. At the time of the data cutoff, the median duration of follow-up of the 32 eligible patients was 6 months (range, 3.2 to 10.5). TCGA level 3 IlluminaHiseq RNAseqV2 data were downloaded from the Broad GDAC Firehose 2015_11_01 run.

### Animals

The animal studies were approved by the Institutional Review Board of Institute of Zoology, Chinese Academy of Sciences. All animal studies were conducted according to protocols approved by the Animal Ethics Committee of the Institute of Zoology, Chinese Academy of Sciences. Female mice C57BL/6, C3H (5-6 weeks old) were purchased from the Vital River Laboratory Animal Technology Co. Ltd. (Beijing, China). A/J mice (5-6 weeks old, female) and homozygous AhR-deficient mice were purchased from the Jackson Laboratory (Bar Harbor, Maine, USA). The A/J mice were exposed to cigarette smoke^31^ generated by DSI’s Buxco Smoke Generator (Buxco, NC, USA) inside a perspex box, at a frequency of 12 cigarettes per day, 5 days per week for 20 days to 6 months. Whole body cigarette smoke exposure per cigarette was 3 minutes followed by a 15-minute period of fresh air. In other experiments, the mice were treated with BaP at 100 mg/kg twice a week for 5 weeks^10^. C57BL/6 mice were numbered, injected with LLC cells (5 × 10^5^) via tail vein, three days after xenograft, the mice were randomized into groups, and treated with vehicle control (coin oil) or ANF at 50, 100, 200 mg/kg/day, or CH223191 at 20 mg/kg/day, for 22 days. NOD/SCID mice were numbered, inoculated with LLC (5 × 10^5^) cells, randomized into groups, and treated with vehicle control or ANF at 200 mg/kg/day for 22 days. Three weeks after the last dose of ANF administration, the mice were anesthetized with the mixture of oxygen/isoflurane inhalation and scanned by microscopic computed tomography (Micro-CT, PerkinElmer, Waltham, MA). Survival of the mice was evaluated from the first day of treatment until death or became moribund, at which time points the mice were euthanized by cervical dislocation. C57BL/6 and B6C3F1 mice were numbered, respectively subcutaneously injected with 5 × 10^5^ MC38 and Ag104Ld cells into their right flank, randomized into groups, and treated intraperitoneally with ANF and/or anti-PD-L1 four times on days 7, 10, 13 and 16^32^. Tumor size was measured every alternate day with electronic caliper and calculated by the formula: volume (mm^3^) = ½ (width)^2^ × length. After 22 days of treatment, the mice were sacrificed by cervical dislocation, tumour tissues were excised, photographed, and subjected to HE and IHC staining.

### Antibodies and reagents

Antibodies used included rabbit anti-human PD-L1 (#13684, Cell Signaling Technology, Beverly, MA, USA; 1:1000 for Western blot), anti-Cleaved Caspase-3 (#9664, Cell Signaling Technology; 1:250 for IHC), Rabbit anti-human AhR (#83200, Cell Signaling Technology; 1:50 for ChIP), goat anti-mouse PD-L1 (#AF1019, R&D, Minneapolis, MN, USA; 10 µg/mL for IHC), anti-Ki67 (#ab15580, Abcam, Cambridge, MA, USA; 1:500 for IHC), TTF1 (#ab76013, Abcam, Cambridge, MA, USA; 1:200 for IHC), anti-β-Actin (#A1978, Sigma, St. Louis, MO, USA; 1:5000 for WB), APC anti-mouse CD3 (#100235, Biolegend, San Diego, CA, USA; 1:20 for flow cytometry), PE/Cy7 anti-mouse CD8a (#100721, Biolegend; 1:20 for flow cytometry), APC/Cy7 anti-mouse CD4 (#100413, Biolegend; 1:20 for flow cytometry), PE anti-mouse CD45 (#103105, Biolegend; 1:20 for flow cytometry), PE/Cy7 anti-mouse PD-L1 (#124313, Biolegend; 1:20 for flow cytometry), PE anti-mouse PD-1 (#135206, Biolegend; 1:20 for flow cytometry), FITC anti-mouse B220 (#103205, Biolegend, San Diego, CA, USA; 1:20 for flow cytometry), In Vivo Plus anti-mouse PD-L1 (#BP0101), In Vivo Plus Rat IgG 2b Isotype Control (Clone: LTF-2, #BP0090) were purchased from BioXcell (West Lebanon, NH, USA). Benzo(a)pyrene (#B1760) and Alpha-Naphthoflavone (ANF; # N5757) were purchased from Sigma.

### Cell culture and RNA extraction

The human normal bronchial epithelial cell line 16HBE (Clonetics, Walkersville, MD), NSCLC line H460, murine LLC (the American Type Culture Collection (ATCC), Manassas, VA, USA), MC38, and Ag104Ld cells were cultured according to recommended protocols^10,52^. The total RNA was isolated using the TRIZOL Reagent (Invitrogen, Frederick, MD, USA) and the phenol-chloroform extraction method according to the manufacturer’s instruction. Total RNA (2 μg) was annealed with random primers at 65 °C for 5 min. The cDNA was synthesized using a 1st-STRAND cDNA Synthesis Kit (Fermentas, Pittsburgh PA, USA). Quantitative real-time PCR was carried out using SYBR Premix ExTaq^TM^ (Takara Biotechnology, Dalian, China). Chromatin immunoprecipitation (ChIP) assay was performed using AhR-immunoprecipitated DNA samples and primers listed in Supplementary Table 2.

### Flow cytometry

Mouse lung cancer tissues were dissected into 2 mm fragments, followed by collagenase IV (0.3%; Sigma) digestion for 20 min at 37 °C. A single-cell suspension was generated through a 200 mm-stainless steel wire mesh. The dissociated cancer cells labeled with indicated cell surface markers were sorted by MoFlo XDP Cell Sorter (Beckman Coulter, Brea, CA, USA), and the data was analyzed on the Summit Software v5.0 (Beckman Coulter). All FACS analyses and sorting were paired with matched isotype control. Dead cells were excluded based on scatter profile.

### Western blotting

Cells were lysed on ice for 30 min in RIPA buffer (50 mM Tris-HCl pH 7.4, 150 mM NaCl, 0.1% SDS, 1% deoxycholate, 1% TritonX-100, 1 mM EDTA, 5 mM NaF, 1 mM sodium vanadate, and protease inhibitors cocktail), and protein extracts were quantitated. Proteins (20 μg) were subjected to 10-15% SDS-PAGE, electrophoresed and transferred to a nitrocellulose membrane. After blocking with 5% non-fat milk in Tris-buffered saline, the membrane was washed and incubated with the indicated primary and secondary antibodies and detected by Luminescent Image Analyzer LSA 4000 (GE, Fairfield, CO, USA). The uncropped and unprocessed scans of Figs. 1h. 2e, and 3l are shown in Supplementary Fig. 6–8.

### Immunofluorescence Microscopy

Cells grown on coverslip (24 mm × 24 mm) were fixed with 4% paraformaldehyde for 15 min, washed with 150 mM glycine in PBS, and permeabilized with 0.3% Triton X-100 in PBS for 20 min at room temperature. After blocking with 5% BSA, the cell smears were incubated with the indicated primary antibodies overnight at 4 °C, washed, and Alexa Flour® 488/647-labeled secondary antibody (life technologies) in PBS was added to the cell smears. Images were taken by a laser scanning confocal microscope (N-STORM, Nikon, Japan).

### Immunohistochemistry analysis

IHC assay was performed using indicated primary antibodies. The formalin-fixed, paraffin-embedded human or mouse lung cancer tissue specimens (5 µm) were deparaffinized through xylene and graded alcohol, and subjected to a heat-induced epitope retrieval step in citrate buffer solution. The sections were then blocked with 5% BSA for 30 min and incubated with indicated antibodies at 4 °C overnight, followed by incubation with secondary antibodies for 90 min at 37 °C. Detection was achieved with 3, 3’-diaminobenzidine (DAB, Zhongshan Golden Bridge Biotechnology Co., Ltd, Beijing, China) and counterstained with hematoxylin, dehydrated, cleared and mounted as in routine processing. The immunoreactivity score was calculated as IRS (0–12) = RP (0–4) × SI (0–3), where RP is the percentage of staining-positive cells and SI is staining intensity.

### Statistical analysis

All statistical analyses were conducted using a GraphPad Prism 5 (GraphPad Software, Inc., La Jolla, CA, USA) and the software SPSS 16.0 for Windows (Chicago, IL, USA). Statistically significant differences were determined by Students *t*-test or Fisher’s exact test. Survival curve for each group was estimated by the Kaplan–Meier method and log-rank test. *P* values less than 0.05 were considered statistically significant in all cases.

### Reporting Summary

Further information on experimental design is available in the Nature Research Reporting Summary linked to this article.

## Supplementary information

Supplementary Information

Reporting Summary

## Data Availability

The microarray data of normal human bronchial cells was downloaded from the Gene Expression Omnibus (http://www.ncbi.nlm.nih.gov/geo/) using datasets under the accession codes GDS1348 and GDS3493. Cancer microarray data was downloaded from the Okayama Lung of the oncomine (the data can also be found at the Gene Expression Omnibus, GSE31210), and the TCGA database using accession code phs000178. All the remaining data supporting the findings of this study are available within this paper and its supplementary information.
