# Author Correction: The Aryl hydrocarbon receptor mediates tobacco-induced PD-L1 expression and is associated with response to immunotherapy

**DOI:** 10.1038/s41467-022-30871-x

**Published:** 2022-06-22

**Authors:** Gui-Zhen Wang, Li Zhang, Xin-Chun Zhao, San-Hui Gao, Li-Wei Qu, Hong Yu, Wen-Feng Fang, Yong-Chun Zhou, Fan Liang, Chen Zhang, Yun-Chao Huang, Zhihua Liu, Yang-Xin Fu, Guang-Biao Zhou

**Affiliations:** 1 State Key Laboratory of Molecular Oncology, National Cancer Center/National Clinical Research Center for Cancer/Cancer Hospital, Chinese Academy of Medical Sciences and Peking Union Medical College, Beijing, 100021 China; 2 State Key Laboratory of Membrane Biology, Institute of Zoology, Chinese Academy of Sciences & University of Chinese Academy of Sciences, Beijing, 100101 China; 3 State Key Laboratory of Oncology in South China; Collaborative Innovation Center for Cancer Medicine; Medical Oncology Department, Sun Yat-Sen University Cancer Center, Guangzhou, 510060 China; 4 School of Chinese Materia Medica, Beijing University of Chinese Medicine, No. 11, Bei San Huan Dong Lu, Beijing, 100029 China; 5 Department of Thoracic Surgery, the Third Affiliated Hospital of Kunming Medical University (Yunnan Tumor Hospital), Kunming, 650106 China; 6 Department of Pathology, University of Texas Southwestern Medical Center, Dallas, TX 75390 USA

Correction to: *Nature Communications* 10.1038/s41467-019-08887-7, published online 8 March 2019.

This Article contains an error in Fig. 1 and the acknowledgments section. In Fig. 1f the DAPI image for the BAP-treated 16HBE cells was incorrect.
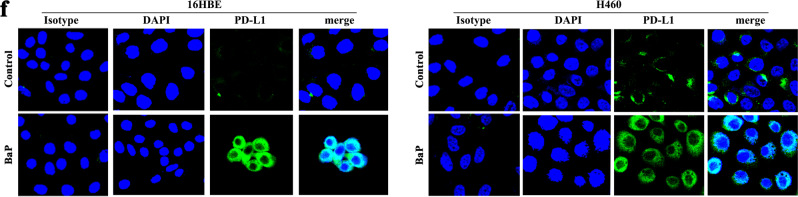


The correct image is shown below.
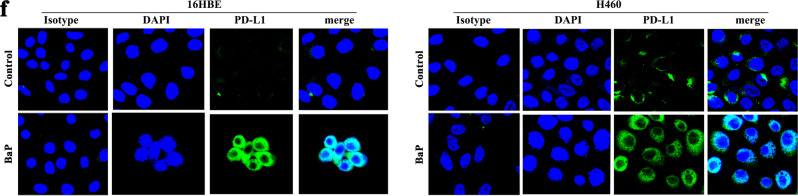


In addition, the acknowledgments section inadvertently omitted mention of a CAMS Innovation Fund for Medical Sciences (CIFMS; No. 2019-I2M-1-003). The correct version of the acknowledgments should read:

This work was supported by the National Natural Science Funds for Distinguished Young Scholar (81425025), the Key Project of the National Natural Science Foundation of China (81830093), the National Key Research and Development Program of China (2016YFC0905500), the National Natural Science Foundation of China (81672765, 81802796), the “Personalized Medicines—Molecular Signature-based Drug Discovery and Development”, Strategic Priority Research Program of the Chinese Academy of Sciences (XDA12010307), and the CAMS Innovation Fund for Medical Sciences (CIFMS; No. 2019-I2M-1-003). The study sponsors had no role in the design of the study; the data collection, analysis, or interpretation; the writing of the article; or the decision to submit for publication.

In addition, this Article was published without describing the number of mice or replicates examined in the figure legends. The following information should have been included in the figure legends.

Figure 1b, d and e results are an average of three biologically independent experiments. Figure 2c, f–h results are representative of tjhree biologically independent replicates.

Figure 2a results are an average of four mice per group, c, d and h results are an average of three mice per group. Figure 2k results are an average of six mice per group. Figure 2n results are an average of three mice per group.

Figure 3a–c, e, g results are an average of three biologically independent experiments. Figure 3h results are an average of six mice per group. Figure 3j: *n* = 10 mice per group. Figure 3k results are an average of three mice per group. Information is also missing on the name of the statistical test and the definition of the error bars in panels Fig. 3b, c, e, g, h, and k. The legend should state; for these panels, Student’s *t-*test, *P < 0.05; **P < 0.01. Error bars, sd.

Figure 6c, l–n results are an average three mice per group.

Supplementary Fig. 3a: *n* = 10 mice per group, and images of four representative mice are shown. Supplementary Fig. 3b-c results are an average of *n* = 10 mice per group. Information is also missing on the definition of the error bars. The legend should state ‘error bars, s.d’.

Supplementary Fig. 4a results are an average of *n* = 10 mice per group. Supplementary Fig. 4b results are an average of *n* = 5 mice per group. Supplementary Fig. 4c results are an average of *n* = 5 mice per group. Supplementary Fig. 4d results are an average of *n* = 3 mice per group, and each experiment was done in triplicate. Supplementary Fig. 4f results are an average of *n* = 3 mice per group, and each experiment was done in triplicate. Supplementary Fig. 4g results are an average of *n* = 10 mice per group. Information is also missing on the name of the statistical test carried out and definition of the error bars. The legend should state for panels a–d, f–g ‘Error bars, sd. Student’s *t*-test, *P < 0.05; **P < 0.01.’

Supplementary Fig. 5a results are an average of *n* = 8 mice per group. Student’s *t-*test, *P < 0.05. Supplementary Fig. 5b results are an average of *n* = 3 mice per group, and the experiments were done in triplicate. Information is also missing on the name of the statistical test carried out. This should stat ‘Student’s *t*-test, **P < 0.01’.

Supplementary Fig. 1 is missing a legend, which should read ‘PD-L1 was detected by quantitative real-time PCR. Three biologically independent experiments were conducted, and each experiment was done in triplicate. Error bars, sd’.

These errors have not been corrected in the PDF and HTML versions of the Article.

