# Supplementary material for: The Aryl hydrocarbon receptor mediates tobacco-induced PD-L1 expression and is associated with response to immunotherapy
Source: Nat Commun. 2019 Mar 8;10:1125. doi: 10.1038/s41467-019-08887-7 (PMC6408580; doi:10.1038/s41467-019-08887-7)
Supplement: Supplementary file 1 — Supplementary Information [file 41467_2019_8887_MOESM1_ESM.pdf]

Supplementary Information

Wang et al.

The Aryl hydrocarbon receptor mediates tobacco-induced PD-L1 expression and is associated with response to immunotherapy

Supplementary Table 1. The 422 genes sequenced in tumor samples harvested before the patients received pembrolizumab.

ABCB1(MDR1)	CDK10	ERCC1	IDH2	MYC	PRKAR1A	SPOP
ABCB4	CDK12	ERCC2	IGF1R	MYCL	PRKCI	SPRY4
ABCC2(MRP2)	CDK4	ERCC3	IGF2	MYCN	PRKDC	SRC
ADH1A	CDK6	ERCC4	IKBKE	MYD88	PRSS1	SRY
ADH1B	CDK8	ERCC5	IKZF1	MYH9	PRSS3	STAG2
ADH1C	CDKN1A	ESR1	IL7R	NAT1	PTCH1	STAT3
AIP	CDKN1B	ETV1	INPP4B	NAT2	PTEN	STK11
AKT1	CDKN1C	ETV4	IRF2	NBN	PTK2	STMN1
AKT2	CDKN2A	EWSR1	JAK1	NCOR1	PTPN11	STT3A
AKT3	CDKN2B	EXT1	JAK2	NF1	PTPN13	SUFU
ALDH2	CDKN2C	EXT2	JAK3	NF2	PTPRD	TEK
ALK	CEBPA	EZH2	JARID2	NFE2L2	QKI	TEKT4
AMER1	CEBPB	FANCA	JUN	NFKBIA	RAC1	TERC
APC	CEBPD	FANCC	KDM5A	NKX2-1	RAC3	TERT
AR	CEP57	FANCD2	KDM6A	NKX2-2	RAD50	TET2
ARAF	CHD4	FANCE	KDR(VEGFR2)	NKX2-4	RAD51	TGFBR2
ARID1A	CHEK1	FANCF	KEAP1	NOTCH1	RAD51C	THADA
ARID1B	CHEK2	FANCG	KIF1B	NOTCH2	RAD51D	TMEM127
ARID2	CLEC2D	FANCL	KIF5B	NOTCH3	RAF1	TMPRSS2
ARID5B	CREBBP	FANCM	KIT	NPM1	RARA	TNFAIP3
ASCL4	CRKL	FAT1	KITLG	NQO1	RARG	TNFRSF11A
ASXL1	CSF1R	FBXW7	KLLN	NRAS	RASGEF1A	TNFRSF14
ATF1	CTCF	FGF19	KMT2A(MLL)	NRG1	RB1	TNFRSF19
ATIC	CTLA4	FGFR1	KMT2B	NSD1	RECQL4	TNFSF11
ATM	CTNNB1	FGFR2	KMT2C	NTRK1	RELN	TOP1
ATR	CUL3	FGFR3	KMT2D(MLL2)	NTRK3	RET	TOP2A
ATRX	CUX1	FGFR4	KRAS	PAK3	RHOA	TP53
AURKA	CXCR4	FH	LHCGR	PALB2	RICTOR	TP63
AURKB	CYLD	FLCN	LMO1	PALLD	RNF43	TPMT
AXIN2	CYP19A1	FLT1(VEGFR1)	LRP1B	PARK2	ROS1	TSC1
AXL	CYP2A13	FLT3	LYN	PARP1	RPTOR	TSC2
BAI3	CYP2A6	FLT4	LZTR1	PARP2	RRM1	TSHR
BAK1	CYP2A7	FOXA1	MAP2K1(MEK1)	PAX5	RUNX1	TTF1
BAP1	CYP2B6*6	FOXP1	MAP2K2(MEK2)	PBRM1	RUNX1T1	TUBB

BARD1	*CYP2C19*2*	*FRG1*	*MAP2K4*	*PDCD1(PD1)*	*RUNX3*	*TUBB2A*
BCL	*CYP2C9*3*	*GATA1*	*MAP3K1*	*PDCD1LG2(PD-L2)*	*SBDS*	*TUBB2B*
BCL2L11(BIM)	*CYP2D6*	*GATA2*	*MAP3K4*	*PDE11A*	*SDC4*	*TUBB3*
BCR	*CYP3A4*4*	*GATA3*	*MAP4K3*	*PDGFRA*	*SDHA*	*TUBB4A*
BIRC3	*CYP3A5*	*GATA4*	*MAX*	*PDGFRB*	*SDHB*	*TUBB4B*
BLM	*DAXX*	*GATA6*	*MCL1*	*PDK1*	*SDHC*	*TUBB6*
BMPR1A	*DDR2*	*GNA11*	*MDM2*	*PGR*	*SDHD*	*TYMS*
BRA	*DENND1A*	*GNA15*	*MDM4*	*PHOX2B*	*SEPT9*	*U2AF1*
BRCA1	*DHFR*	*GNAQ*	*MECOM*	*PIK3C3*	*SETBP1*	*UGT1A1*
BRCA2	*DHFRL1*	*GNAS*	*MED12*	*PIK3CA*	*SETD2*	*VEGFA*
BRD4	*DICER1*	*GRIN2A*	*MEF2B*	*PIK3R1*	*SF3B1*	*VHL*
BRIP1	*DNMT3A*	*GRM3*	*MEN1*	*PIK3R2*	*SGK1*	*WAS*
BTG2	*DPYD*	*GRM8*	*MET*	*PKHD1*	*SLC34A2*	*WISP3*
BT	*DUSP2*	*GSTM1*	*MGMT*	*PLAG1*	*SLC7A8*	*WRN*
BUB1B	*EGFR*	*GSTM4*	*MIT*	*PLK1*	*SMAD2*	*WT1*
c11orf30	*EML4*	*GSTM5*	*MLH1*	*PMS1*	*SMAD3*	*XPA*
CASP8	*EP300*	*GSTP1*	*MLH3*	*PMS2*	*SMAD4*	*XPC*
CB	*EPAS1*	*GSTT1*	*MLLT1*	*POLD1*	*SMAD7*	*XRCC1*
CBL	*EPCAM*	*HDAC2*	*MLLT3*	*POLD3*	*SMARCA*	*YAP1*
CC2D2B	*EPHA2*	*HDAC9*	*MLLT4*	*POLE*	*SMARCB*	*ZNF2*
CCND1	*EPHA3*	*HG*	*MPL*	*POLH*	*SMO*	*ZNF217*
CCNE1	*EPHA5*	*HLA-A*	*MRE11A*	*POT1*	*SOS1*	*ZNF703*
CD274(PD-L1)	*EPHB2*	*HNF1A*	*MSH2*	*PPP2R1A*	*SOX1*	
CD74	*ERBB2(HER2*	*HNF1B*	*MSH6*	*PRDM1*	*SOX14*	
CDA	*ERBB2IP*	*HRAS*	*MTHFR*	*PRF1*	*SOX2*	
CDC73	*ERBB3*	*HSD3B1*	*MTOR*	*PRKACA*	*SOX21*	
CDH1	*ERBB4*	*IDH1*	*MUTYH*	*PRKACG*	*SOX3*	

Supplementary Table 2. Primers used in the study.

Target	Forward primer (5'→3')	Reverse primer (5'→3')
**HUMAN**		
GAPDH	GAAGGTGAAGGTCGGAGTC	GAAGATGGTGATGGGATTTC
Pd-l1	AGTGGTAAGACCACCACCACCAAT	TCATTTGGAGGATGTGCCAGAGGT
**MOUSE**		
GAPDH	AGTATGACTCCACTCACGGCAA	TCTCGCTCCTGGAAGATGGT
Pd-l1	GCTCCAAAGGACTTGTACGTG	TGATCTGAAGGGCAGCATTTC
TNFa	CCTCCACTTGGTGGTTTGCTA	CAGACCCTCACACTCAGATCATCT
IFNγ	TCAAGTGGCATAGATGTGGAAGAA	TGGCTCTGCAGGATTTTCATG
**ChIP**		
Pd-l1 (region 1)	CTGAAAGCTTCCGCCGATT	CTACCTGCAGGCGGACAGA
Pd-l1 (region 2)	CCCATTCACTAACCCAAAGCT	AAAAGAACTTCCCATCCCGA
**siRNA**	**Sequence 1 (5'→3')**	**Sequence 2 (5'→3')**
siAhR	GGAUUAAAUUAGUUUGUGAdTdT	UCACAAACUAAUUUAAUCCdAdA

Supplementary figures and legends

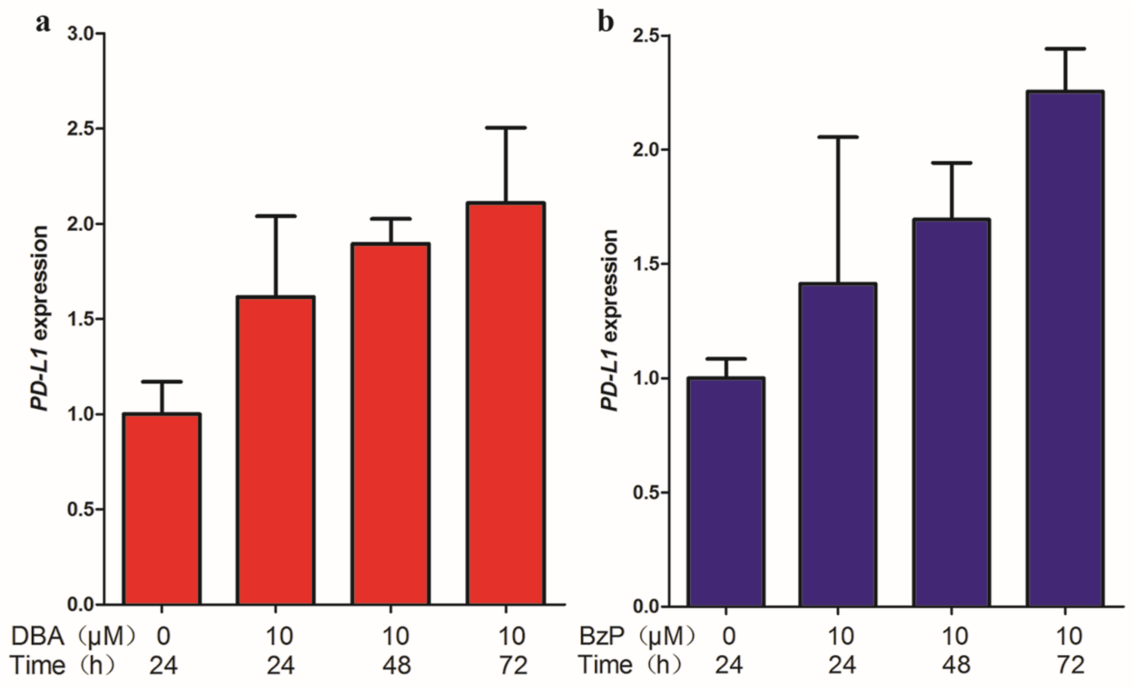

Supplementary Figure 1. The effects of DBA (a) and BzP (b) on the expression *PD-L1* in 16HBE cells.

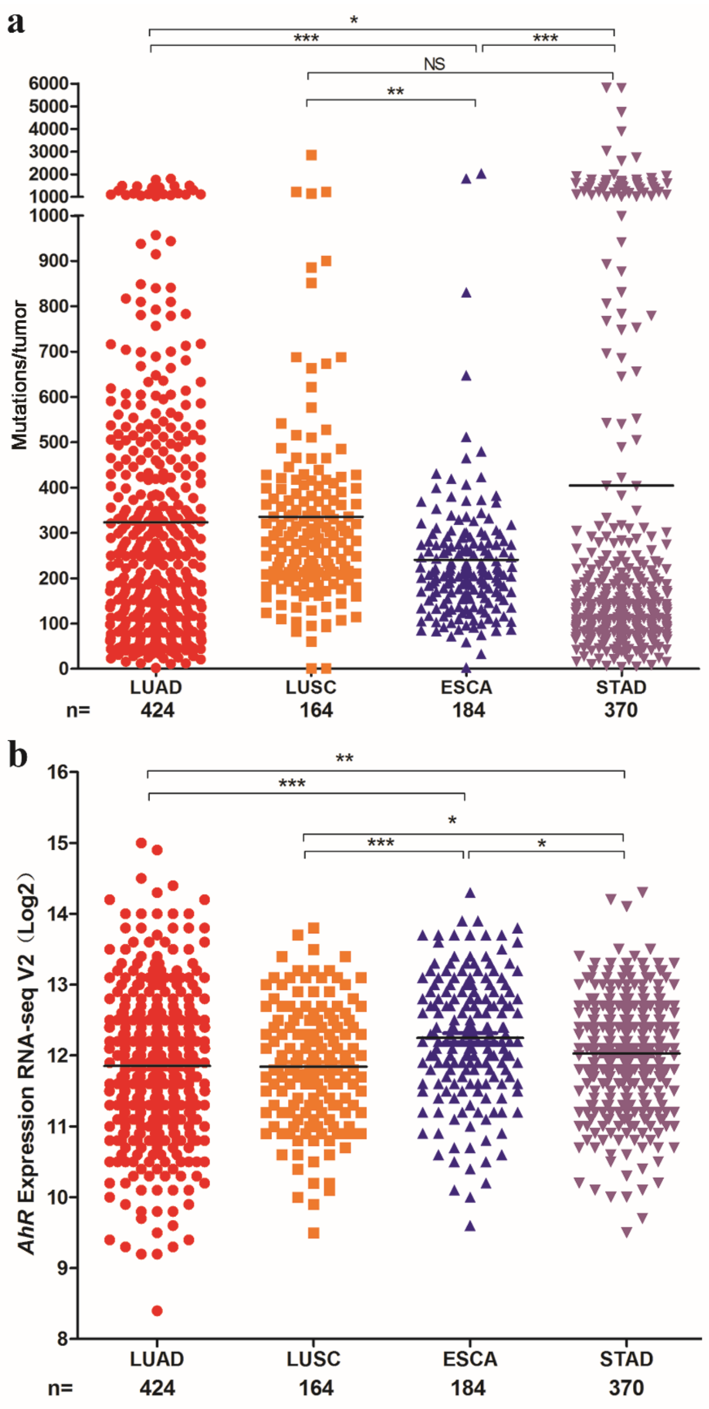

Supplementary Figure 2. AhR expression levels are not associated with mutation loads of the patients. Data are from TCGA datasets. (a) Mutation loads of patients with LUAD, LUSC, esophageal carcinoma (ESCA), and stomach adenocarcinoma (STAD). (b) *AhR* expression levels of the patients.

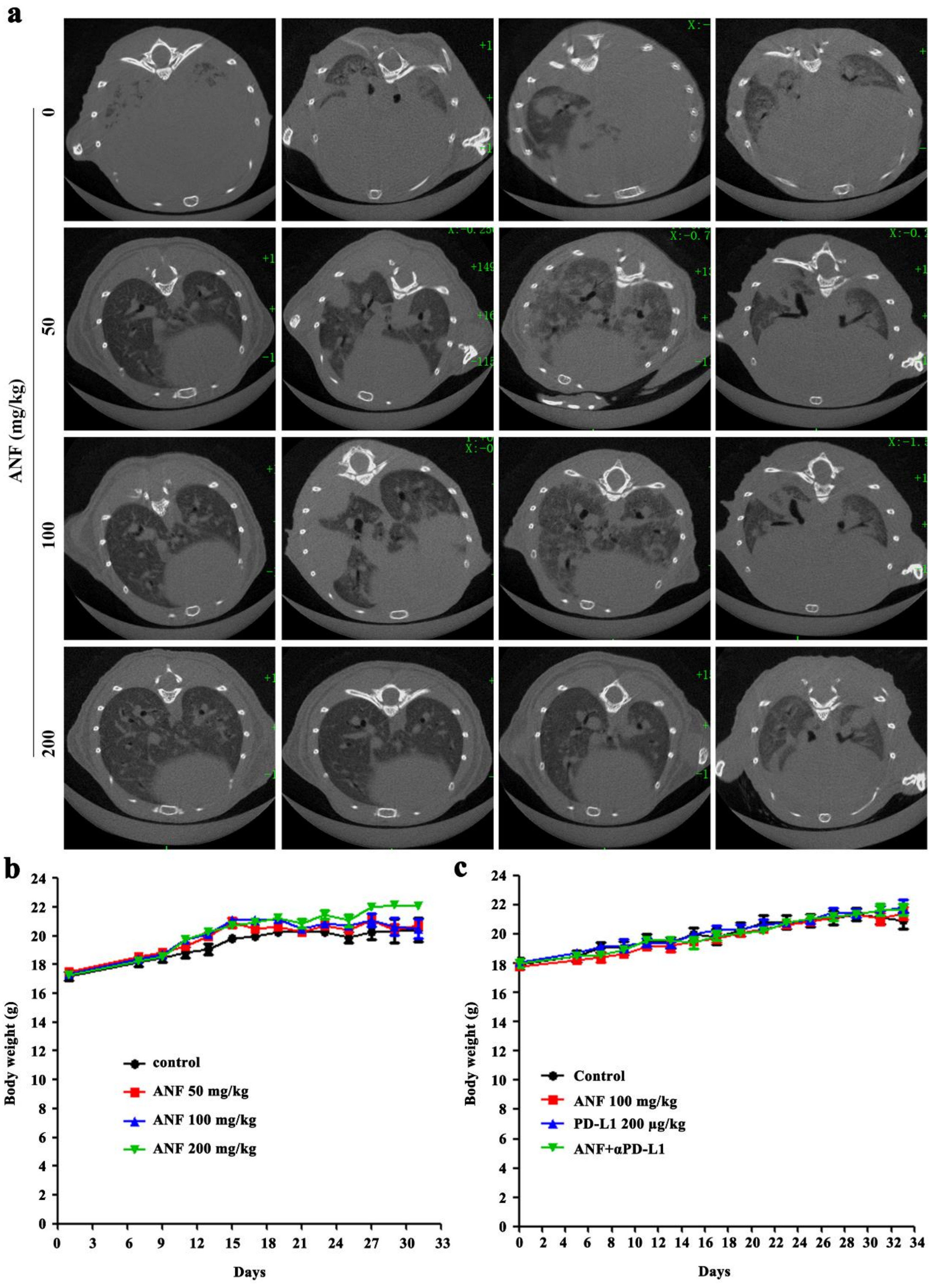

Supplementary Figure 3. AhR inhibitor exhibits anti-lung cancer activity and low toxicity in C57BL/6. mice. (a) The C57BL/6 mice were intravenously injected with LLC cells, and 3 days later randomized to receive vehicle or ANF treatment. Micro-CT scanning images of the mice are shown. (b) Treatment of ANF at 50 to 200 mg/kg did not affect the body weight of

C57BL/6 mice. (c) Treatment of ANF and/or anti-PD-L1 antibody did not significantly affect the body weight of the mice.

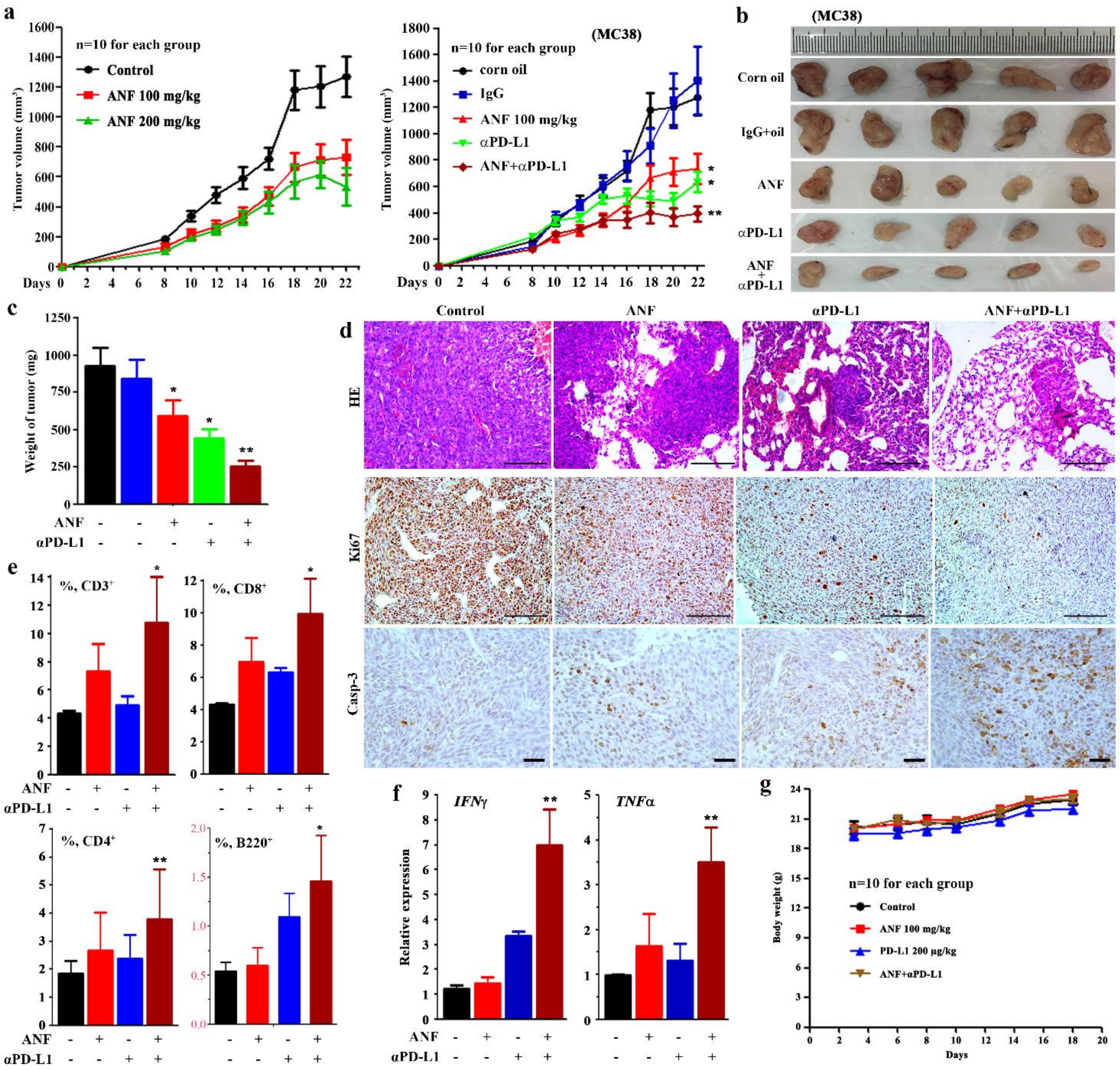

Supplementary Figure 4. Effects of AhR inhibitor on colon cancer murine model. (a) C57BL/6 mice were subcutaneously inoculated with 5×10^5 MC38 cells, and then treated with ANF and/or anti-PD-L1 antibody. Tumor volume was estimated every two days. (b) Images of xenograft tumors obtained from the mice. (c) Weight of xenograft tumors obtained from the mice. (d) HE staining and IHC analyses of lung sections of the mice. (e) Flow cytometry

analysis of CD45$^+$CD3$^+$, CD8$^+$, CD4$^+$, and B220$^+$ cells in the lung tissues. (f) The expression of *IFNγ* and *TNFα* in the lung tissues was detected by real-time PCR. (g) Body weight of C57BL/6 mice treated with ANF and/or anti-PD-L1 antibody. Error bars, sd.

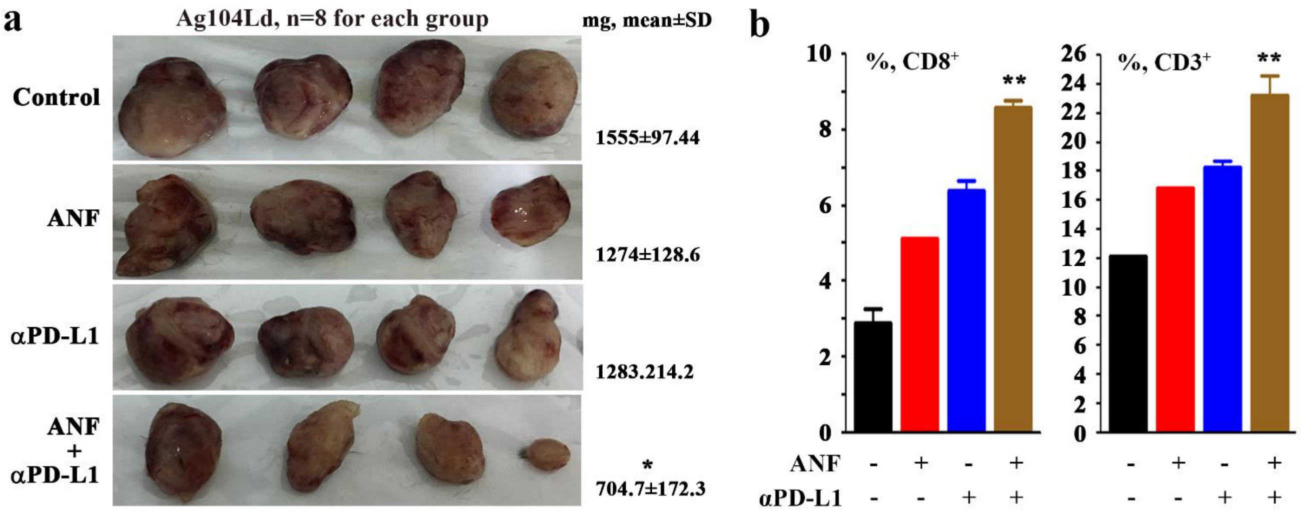

Supplementary Figure 5. The therapeutic efficacy of AhR inhibition in fibrosarcoma murine model. (a) B6C3F1 mice were subcutaneously injected with Ag104Ld (5×10^5) cells and treated with ANF and/or an anti-PD-L1 antibody. Images showed xenograft tumors obtained from the mice. (b) Flow cytometry analysis of CD8$^+$ and CD3$^+$ cells in the lung tissues. Error bars, sd.

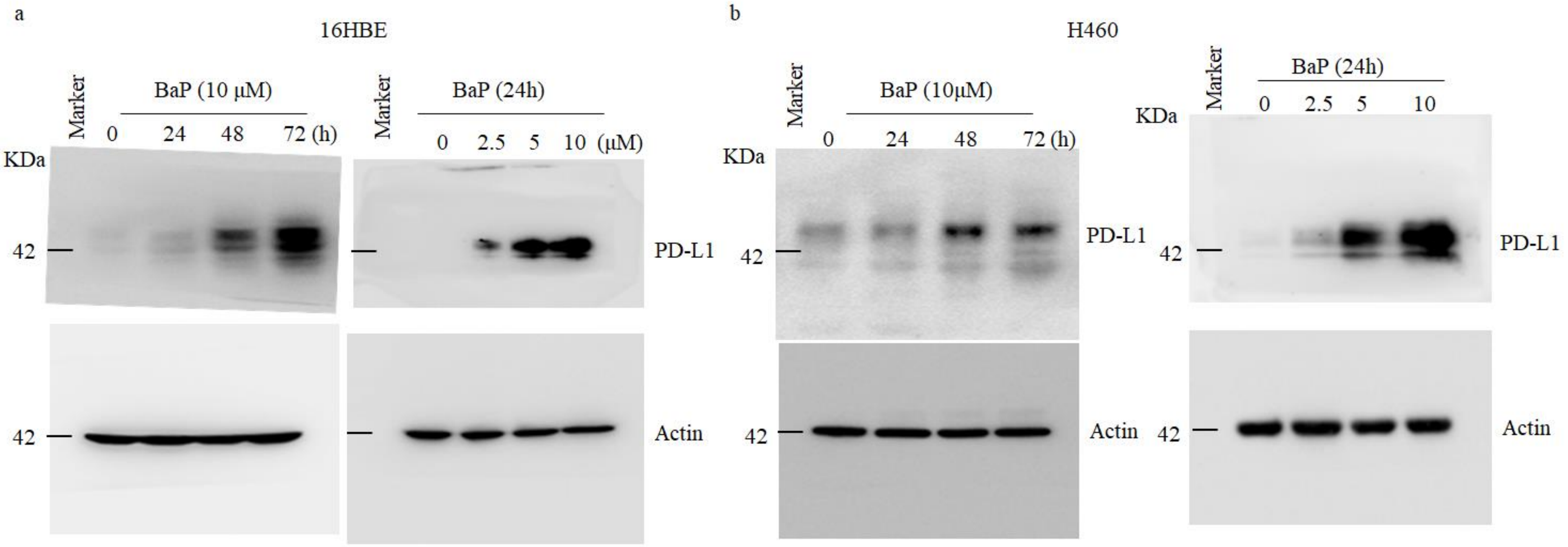

Supplementary Fig. 6. Uncropped and unprocessed scans of Fig. 1h.

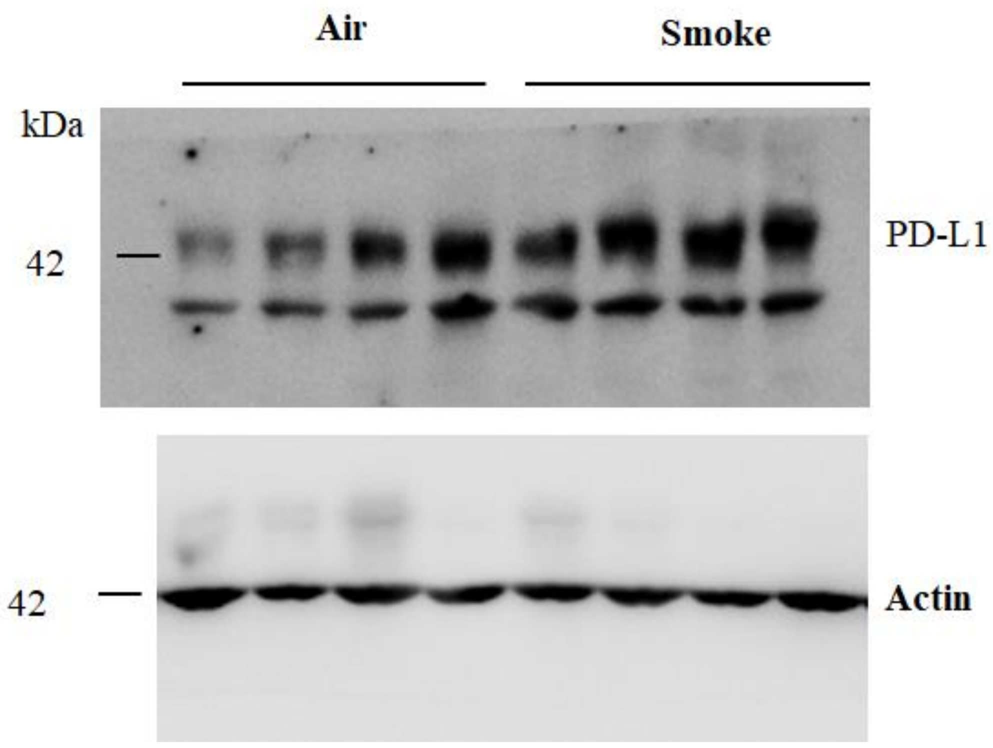

Supplementary Fig. 7. Uncropped and unprocessed scans of Fig. 2e.

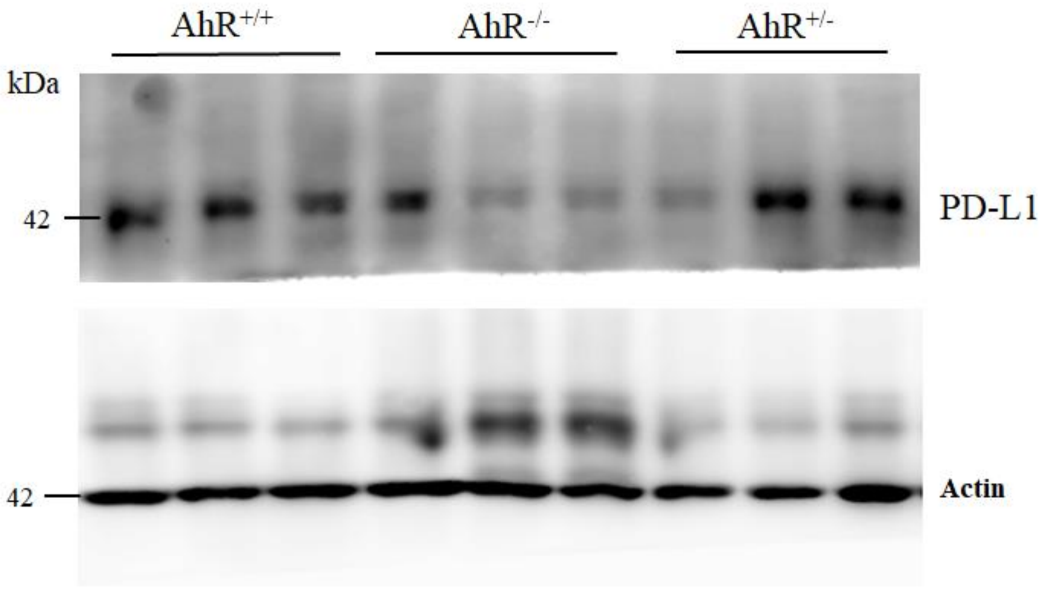

Supplementary Fig. 8. Uncropped and unprocessed scans of Fig. 3l.
