# Supplementary material for: The Aryl hydrocarbon receptor mediates tobacco-induced PD-L1 expression and is associated with response to immunotherapy
Source: Nat Commun. 2019 Mar 8;10:1125. doi: 10.1038/s41467-019-08887-7 (PMC6408580; doi:10.1038/s41467-019-08887-7)
Supplement: Supplementary file 2 — Reporting Summary [file 41467_2019_8887_MOESM2_ESM.pdf]

nature research

Corresponding author(s): Guang-Biao Zhou

Last updated by author(s): Jan 5, 2019

Reporting Summary

Nature Research wishes to improve the reproducibility of the work that we publish. This form provides structure for consistency and transparency in reporting. For further information on Nature Research policies, see Authors & Referees and the Editorial Policy Checklist.

Statistics

For all statistical analyses, confirm that the following items are present in the figure legend, table legend, main text, or Methods section.

n/a	Confirmed	
☐	☒	The exact sample size (*n*) for each experimental group/condition, given as a discrete number and unit of measurement
☐	☒	A statement on whether measurements were taken from distinct samples or whether the same sample was measured repeatedly
☐	☒	The statistical test(s) used AND whether they are one- or two-sided<br>*Only common tests should be described solely by name; describe more complex techniques in the Methods section.*
☐	☒	A description of all covariates tested
☐	☒	A description of any assumptions or corrections, such as tests of normality and adjustment for multiple comparisons
☐	☒	A full description of the statistical parameters including central tendency (e.g. means) or other basic estimates (e.g. regression coefficient) AND variation (e.g. standard deviation) or associated estimates of uncertainty (e.g. confidence intervals)
☒	☐	For null hypothesis testing, the test statistic (e.g. *F*, *t*, *r*) with confidence intervals, effect sizes, degrees of freedom and *P* value noted<br>*Give P values as exact values whenever suitable.*
☒	☐	For Bayesian analysis, information on the choice of priors and Markov chain Monte Carlo settings
☒	☐	For hierarchical and complex designs, identification of the appropriate level for tests and full reporting of outcomes
☒	☐	Estimates of effect sizes (e.g. Cohen's *d*, Pearson's *r*), indicating how they were calculated

Our web collection on statistics for biologists contains articles on many of the points above.

Software and code

Policy information about availability of computer code

Data collection	n/a
Data analysis	n/a

For manuscripts utilizing custom algorithms or software that are central to the research but not yet described in published literature, software must be made available to editors/reviewers. We strongly encourage code deposition in a community repository (e.g. GitHub). See the Nature Research guidelines for submitting code & software for further information.

Data

Policy information about availability of data

All manuscripts must include a data availability statement. This statement should provide the following information, where applicable:

- Accession codes, unique identifiers, or web links for publicly available datasets
- A list of figures that have associated raw data
- A description of any restrictions on data availability

All the data supporting the findings of this study are available within this paper and its supplementary information.

Field-specific reporting

Please select the one below that is the best fit for your research. If you are not sure, read the appropriate sections before making your selection.

☒ Life sciences ☐ Behavioural & social sciences ☐ Ecological, evolutionary & environmental sciences

For a reference copy of the document with all sections, see nature.com/documents/nr-reporting-summary-flat.pdf

Life sciences study design

All studies must disclose on these points even when the disclosure is negative.

Sample size	Two settings of patient samples were tested: n=62 for line 142, n=37 for line 185. These sample sizes have the power to detect the difference between the groups.
Data exclusions	No samples or animals were excluded from the analysis
Replication	Cellular experiments were repeated three times; in vivo studies used animals at numbers having the power to test the difference; the mice were exposed to cigarette smoke or BaP to confirm the effects on PD-L1 expression; two AhR inhibitors were used to confirm the effects of AhR inhibitor on murine lung cancer; two settings of patient samples were tested: n=62 for line 142, n=37 for line 185. These sample sizes have the power to ensure the reproducibility.
Randomization	The mice were numbered, intravenously or subcutaneously injected with LLC cells, randomized into groups, and treated with vehicle control or indicated regimens, for indicated time period.
Blinding	Blinding was not used in this study, because the purpose of the clinical study was to test the efficacy of pembrolizumab in Chinese lung cancer patients.

Reporting for specific materials, systems and methods

We require information from authors about some types of materials, experimental systems and methods used in many studies. Here, indicate whether each material, system or method listed is relevant to your study. If you are not sure if a list item applies to your research, read the appropriate section before selecting a response.

Materials & experimental systems

n/a	Involved in the study	
☐	☒	Antibodies
☐	☒	Eukaryotic cell lines
☒	☐	Palaeontology
☐	☒	Animals and other organisms
☐	☒	Human research participants
☐	☒	Clinical data

Methods

n/a	Involved in the study	
☒	☐	ChIP-seq
☐	☒	Flow cytometry
☒	☐	MRI-based neuroimaging

Antibodies

Antibodies used	Rabbit anti-human PD-L1(#13684, Cell Signaling Technology, Beverly, MA, USA), anti-Cleaved Caspase-3 (#9664, Cell Signaling Technology, Beverly, MA, USA), Rabbit anti-human AhR (#83200, Cell Signaling Technology, Beverly, MA, USA), goat anti-mouse PD-L1 (#AF1019, R&D, Minneapolis, MN, USA), anti-Ki67 (#ab15580, Abcam, Cambridge, MA, USA), TTF1 (#ab76013, Abcam, Cambridge, MA, USA), anti-β-Actin (#A1978, Sigma, St. Louis, MO, USA), APC anti-mouse CD3 (#100235), PE/Cy7 anti-mouse CD8a (#100721), APC/Cy7 anti-mouse CD4 (#100413), PE anti-mouse CD45 (#103105), PE/Cy7 anti-mouse PD-L1 (#124313), PE anti-mouse PD-1 (#135206), FITC anti-mouse B220 (#103205, Biolegend, San Diego, CA, USA)
Validation	Documents certify that the products have met the quality control standards defined by the manufacturers.

Eukaryotic cell lines

Policy information about cell lines

Cell line source(s)	Cell line sources are listed in page 17.
Authentication	The lines were authenticated by short tandem repeat (STR) profiling.
Mycoplasma contamination	The cell lines were not tested for mycoplasma contamination.
Commonly misidentified lines (See ICLAC register)	n/a

Animals and other organisms

Policy information about studies involving animals; ARRIVE guidelines recommended for reporting animal research

Laboratory animals	Species: C57BL/6, C3H, A/J, NOD/SCID; sex: both; age: 4-6 weeks old.
Wild animals	This study did not involve wild animal.
Field-collected samples	The study did not involve samples collected from the field.
Ethics oversight	The study was approved by the local research ethics committees of the Cancer Hospital, Sun Yat-Sen University, and the Third Affiliated Hospital of Kunming Medical University (Yunnan Tumor Hospital). The animal studies were approved by the Institutional Review Board of Institute of Zoology, Chinese Academy of Sciences. All animal studies were conducted according to protocols approved by the Animal Ethics Committee of the Institute of Zoology, Chinese Academy of Sciences.

Note that full information on the approval of the study protocol must also be provided in the manuscript.

Human research participants

Policy information about studies involving human research participants

Population characteristics	See above
Recruitment	37 Chinese lung cancer patients were treated with pembrolizumab.
Ethics oversight	The study was approved by the Cancer Hospital, Sun Yat-Sen University.

Note that full information on the approval of the study protocol must also be provided in the manuscript.

Clinical data

Policy information about clinical studies

All manuscripts should comply with the ICMJE guidelines for publication of clinical research and a completed CONSORT checklist must be included with all submissions.

Clinical trial registration	Tumor tissues of patients treated with pembrolizumab were tested for the expression of AhR and PD-L1. This study did not involve survival and other important information. For the trial registration number, we contacted the company to have their thoughts. However, they said that the trial is still ongoing to see the overall survival of the patients. They said that they prefer to release the trial registration number when the survival data are obtained. So I prefer not to release the number at this stage and hope you can understand this. Thanks a lot.
Study protocol	Participants receive pembrolizumab 2 mg/kg administered intravenously every 3 weeks for up to 35 administrations.
Data collection	The expression of AhR and PD-L1 in tumor tissues of the patients, and the responses of the patients to pembrolizumab.
Outcomes	The association between expression of AhR and PD-L1 in tumor tissues and the responses of the patients to pembrolizumab.

Flow Cytometry

Plots

Confirm that:

☒ The axis labels state the marker and fluorochrome used (e.g. CD4-FITC).

☒ The axis scales are clearly visible. Include numbers along axes only for bottom left plot of group (a 'group' is an analysis of identical markers).

☒ All plots are contour plots with outliers or pseudocolor plots.

☒ A numerical value for number of cells or percentage (with statistics) is provided.

Methodology

Sample preparation	Fresh tumor samples and counterpart normal lung tissues, or tumor biopsy specimens were collected. The mice were euthanized by cervical dislocation when they became moribund, lungs were isolated, and cancer tissues were harvested.
Instrument	The mice were anesthetized with mixture of oxygen/isoflurane inhalation and scanned by microscopic computed tomography cro-CT, PerkinElmer, Waltham, MA).
Software	The Summit Software v5.0 (Beckman Coulter)
Cell population abundance	n/a

Gating strategy

Cells were gated based on forward and side scatter plots, only avoiding debris and aggregates and no extensive gating strategy.

☒ Tick this box to confirm that a figure exemplifying the gating strategy is provided in the Supplementary Information.
